# Comparative effectiveness of different primary vaccination courses on mRNA-based booster vaccines against SARs-COV-2 infections: a time-varying cohort analysis using trial emulation in the Virus Watch community cohort

**DOI:** 10.1093/ije/dyad002

**Published:** 2023-01-19

**Authors:** Vincent Grigori Nguyen, Alexei Yavlinsky, Sarah Beale, Susan Hoskins, Thomas E Byrne, Vasileios Lampos, Isobel Braithwaite, Wing Lam Erica Fong, Ellen Fragaszy, Cyril Geismar, Jana Kovar, Annalan M D Navaratnam, Parth Patel, Madhumita Shrotri, Sophie Weber, Andrew C Hayward, Robert W Aldridge

**Affiliations:** Institute of Health Informatics, University College London, London, UK; Institute of Epidemiology and Health Care, University College London, London, UK; Institute of Health Informatics, University College London, London, UK; Institute of Health Informatics, University College London, London, UK; Institute of Epidemiology and Health Care, University College London, London, UK; Institute of Epidemiology and Health Care, University College London, London, UK; Institute of Health Informatics, University College London, London, UK; Department of Computer Science, University College London, London, UK; Institute of Health Informatics, University College London, London, UK; Institute of Health Informatics, University College London, London, UK; Institute of Health Informatics, University College London, London, UK; Department of Infectious Disease Epidemiology, London School of Hygiene and Tropical Medicine, London, UK; Institute of Health Informatics, University College London, London, UK; Institute of Epidemiology and Health Care, University College London, London, UK; Institute of Health Informatics, University College London, London, UK; Institute of Health Informatics, University College London, London, UK; Institute of Health Informatics, University College London, London, UK; Institute of Health Informatics, University College London, London, UK; Institute of Epidemiology and Health Care, University College London, London, UK; Institute of Health Informatics, University College London, London, UK

**Keywords:** COVID-19, SARS-CoV-2, mRNA booster vaccination, trial emulation, time-varying confounding-by-indication

## Abstract

**Background:**

The Omicron B.1.1.529 variant increased severe acute respiratory syndrome coronavirus 2 (SARS-CoV-2) infections in doubly vaccinated individuals, particularly in the Oxford–AstraZeneca COVID-19 vaccine (ChAdOx1) recipients. To tackle infections, the UK’s booster vaccination programmes used messenger ribonucleic acid (mRNA) vaccines irrespective of an individual’s primary course vaccine type, and prioritized the clinically vulnerable. These mRNA vaccines included the Pfizer–BioNTech COVID-19 vaccine (BNT162b2) the Moderna COVID-19 vaccine (mRNA-1273). There is limited understanding of the effectiveness of different primary vaccination courses on mRNA booster vaccines against SARs-COV-2 infections and how time-varying confounders affect these evaluations.

**Methods:**

Trial emulation was applied to a prospective community observational cohort in England and Wales to reduce time-varying confounding-by-indication driven by prioritizing vaccination based upon age, vulnerability and exposure. Trial emulation was conducted by meta-analysing eight adult cohort results whose booster vaccinations were staggered between 16 September 2021 and 05 January 2022 and followed until 23 January 2022. Time from booster vaccination until SARS-CoV-2 infection, loss of follow-up or end of study was modelled using Cox proportional hazard models and adjusted for age, sex, minority ethnic status, clinically vulnerability and deprivation.

**Results:**

A total of 19 159 participants were analysed, with 11 709 ChAdOx1 primary courses and 7450 BNT162b2 primary courses. Median age, clinical vulnerability status and infection rates fluctuate through time. In mRNA-boosted adults, 7.4% (*n* = 863) of boosted adults with a ChAdOx1 primary course experienced a SARS-CoV-2 infection compared with 7.7% (*n* = 571) of those who had BNT162b2 as a primary course. The pooled adjusted hazard ratio (aHR) was 1.01 with a 95% confidence interval (CI) of: 0.90 to 1.13.

**Conclusion:**

After an mRNA booster dose, we found no difference in protection comparing those with a primary course of BNT162b2 with those with a ChAdOx1 primary course. This contrasts with pre-booster findings where previous research shows greater effectiveness of BNT162b2 than ChAdOx1 in preventing infection.

Key Messages

- Analyses of vaccination should consider the time-varying confounding-by-indication induced by the priority distribution of vaccines based upon age and clinical vulnerability.
- Trial emulation, particularly cohort staggering through time, is an appropriate method to reduce time-varying confounding-by-indication, as it compares similar individuals who experienced similar public health exposures and SARS-CoV-2 reproduction rates at the time of vaccination and throughout follow-up.
- A booster dose with an mRNA vaccine leads to similar protection against SARS-CoV-2, regardless of the vaccine used for the primary vaccination series, when analysed using trial emulation.

## Introduction

England and Wales experienced an increase in severe acute respiratory syndrome coronavirus 2 (SARS-CoV-2) infections in individuals who received two doses of vaccine. This increase in infection rates is partially attributable to waning vaccine protection and the emergence of the variant of concern, B.1.1.529 (Omicron) which has mutations leading to partial immune escape from prior infection or vaccination.^1^ To tackle the growth in infections, the UK accelerated the delivery of booster vaccines to those who received two doses of coronavirus disease 2019 (COVID-19) vaccinations, with a gap of 3 months between the second dose of the primary series and the first booster dose.

Our previous analysis found a difference in SARS-CoV-2 infection rates between the two dominant vaccines, the Oxford–AstraZeneca COVID-19 vaccine (ChAdOx1) and the Pfizer–BioNTech COVID-19 vaccine (BNT162b2) in England and Wales, with those receiving ChAdOx1 as their primary course having a 35% increased risk of SARS-CoV-2 infection with a hazard ratio (HR) of 1.35 and a 95% confidence interval (CI) of 1.15–1.58 within 315 days following first vaccination.^2^ Our findings are consistent with previous work which demonstrated the difference in peak Spike-antibody levels (the primary antibody stimulated by vaccination-related inoculation) based upon vaccine type, where BNT162b2 produced Spike-antibody levels an order of magnitude higher than ChAdOx1 after two doses.^3^ Due to the differences in: vaccine effectiveness in preventing SARS-CoV-2 infections; enhancing antibody level; and data from recently conducted randomized controlled trials examining safety and immunogenicity of seven COVID-19 vaccines as a booster dose^4^; messenger ribonucleic acid (mRNA)-based vaccines, such as BNT162b2 or the Moderna COVID-19 vaccine (mRNA-1273), were chosen for the booster dose in the UK to tackle further waves of infection.^4^

Following the use of BNT162b2 or mRNA-1273 as booster doses in the UK, research from the United Kingdom Health Security Agency (UKHSA) has demonstrated similar effectiveness between primary vaccine courses after mRNA boosters, using test-negative study designs.^5^ Test-negative designs are well suited to reducing biases related to test-seeking behaviour,^6^ but are subject to temporal confounding where timing (i.e. the date) of vaccination is influenced by risk factors for severe infection such as age, clinical vulnerability and health care work-related exposure.^7^ Vaccine effectiveness estimates from test-negative designs will also be biased if a covariate is associated with both vaccination and the outcome of interest or if vaccination is associated with seeking health care.^8^^,^^9^ To protect the most vulnerable and exposed to SARs-CoV-2, the UK’s strategy prioritized booster vaccination rolled out based upon age, clinical vulnerability and exposure to the virus (for example, front-line health care workers). In addition to variation in timing of booster vaccinations according to risk factors, there are substantial variations in levels of infection and intensity of control measures over time, which complicate the task of controlling for time-varying confounding.

In this study, we aim to apply trial emulation techniques developed by Hernán and Robins^10^ to tackle time-varying confounding by indication. Following Hernán *et al.*’s recommendations to overcome time-varying confounding, we use an eligibility criterion that removes those who are likely to have protection from SARS-CoV-2 (e.g. through prior natural infection) and stagger our cohort based upon vaccination date. Staggering a single cohort into multiple cohorts aims to produce cohorts that are homogeneous in terms of the eligibility criteria that had allowed them to be vaccinated at that point in time. Using staggered cohorts allows similar individuals to have similar follow-up periods and, more importantly, to experience the same COVID-19 public health policies and SARS-CoV-2 reproduction rates at the time of vaccination and throughout their follow-up period. This approach aims to control for the UK’s booster prioritization schedule, but could also mitigate the effects of unmeasured time-varying confounders at the community level, including the introduction of new SARS-CoV-2 variants. Therefore, this approach appropriately accounts for ‘time zero’ (start of follow-up from the booster dose) as it avoids comparisons between individuals who experienced different public health policies and SARS-CoV-2 reproduction rates through time. Our objective is to use trial emulation to appropriately estimate the comparative effectiveness of receiving different primary vaccine courses (ChAdOx1 or BNT162b2) in addition to an mRNA (BNT162b2 or mRNA-1273) booster vaccine against SARs-CoV-2 infections, in a general population community cohort. Therefore, our possible vaccination histories include the following combinations: a primary course of ChAdOx1 with a booster dose of BNT162b2 or mRNA-1273; and a primary course of BNT162b2 with a booster dose of BNT162b2 or mRNA-1273.

## Method

### Study design and setting

The study design used prospective observational data from the Virus Watch Cohort and applied a target trial emulation study design—detailed descriptions of the target trial emulation can be found in Table 1. The Virus Watch cohort has been described previously^11^; briefly, households were recruited starting in mid-June 2020, aimed at creating a representative cohort of England and Wales (see Supplementary Table S1, available as Supplementary data at *IJE* online, for a sociodemographic comparison of the Virus Watch cohort with the Office of National Statistics breakdown for England and Wales). To rapidly recruit participants at the start of the pandemic, we used a range of methods aimed at creating a representative cohort of England and Wales. We used the Royal Mail Post Office Address File to generate a random list of residential address lists that were sent recruitment postcards (*n* = 3914), we placed social media advertisements on Facebook and Twitter (*n* = 18 594) and sent Short Message Service text messages (*n* = 11 151) and letters to participants and households from their general practitioners (*n *= 3803).

**Table 1:** Details of the trial emulation framework used to conceptualize the observational study as a controlled trial

	Ideal randomised controlled trial	Trial emulation
Eligibility criteria	At least 18 years old No prior severe acute respiratory syndrome coronavirus 2 (SARS-CoV-2) infection Two doses of the SARs-CoV-2 vaccine	At least 18 years old when vaccinated No recorded SARS-CoV-2 infection prior to booster vaccination date, defined using: polymerase chain reaction (PCR) test, lateral flow test (LFT), nucleocapsid antibodies and Spike protein before 2021 Two doses of the SARs-CoV-2 vaccine
Recruitment period	16 September 2021 to 05 January 2022	16 September 2021 to 05 January 2022 split by 14-day intervals: Cohort 1: 16 September 2021 to 29 September 2021 Cohort 2: 30 September 2021 to 13 October 2021 Cohort 3: 14 October 2021 to 27 October 2021 Cohort 4: 28 October 2021 to 10 November 2021 Cohort 5: 11 November 2021 to 24 November 2021 Cohort 6: 25 November 2021 to 08 December 2021 Cohort 7: 09 December 2021 to 22 December 2021 Cohort 8: 23 December 2021 to 05 January 2022
Follow-up duration	From 16 September 2021 to 23 January 2022	From recorded booster vaccination date until 23 January 2022
Outcome	Positive PCR test for SARS-CoV-2 Positive LFT for SARS-CoV-2	Positive PCR test for SARS-CoV-2 (self-reported or linked data) Positive LFT for SARS-CoV-2 (self-reported or linked data)
Treatments to be compared	Booster dose with primary course of the Oxford-AstraZeneca vaccine (ChAdOx1) Booster dose with a primary course of the Pfizer-BioNTech vaccine (BNT162b2)	Booster dose with primary course of ChAdOx1 Booster dose with a primary course of BNT162b2
Estimand	Intention to treat based upon primary course	Intention to treat based upon primary course
Analysis plan	Survival analysis (Kaplan–Meier estimator)	Survival analysis (pooled multivariable Cox proportional hazard models)

By February 2022, 58 566 individuals in 28 495 households had registered to take part. Participants completed weekly online surveys reporting symptoms, SARS-CoV-2 swab test results and vaccinations. From autumn 2020, Virus Watch included a programme of nasopharyngeal swab sample collection and blood collection via venepuncture or finger-prick sampling in a subset of 10 000 participants in research clinics. From March 2021, blood samples were self-collected by participants using an at-home capillary blood sample collection kit, manufactured by the company Thriva [https://thriva.co/]. Completed kits were returned by participants using pre-paid envelopes and priority postage boxes to United Kingdom Accreditation Service-accredited laboratories, for serological testing using Roche’s Elecsys Anti-SARS-CoV-2 assays targeting total immunoglobulin (Ig) to the nucleocapsid (N) protein or to the receptor-binding domain in the S1 subunit of the Spike protein (S) (Roche Diagnostics, Basel, Switzerland).

### Participants

Participants were eligible for the current analysis if they had a recorded third (booster) COVID-19 vaccination between 16 September 2021 and 05 January 2022. Participants must have had a primary COVID-19 vaccination dose recorded as of either ChAdOx1 or BNT162b2. As the UK vaccination programme only included children under 18 years of age in the second half of 2021, participants under 18 years old were excluded from these analyses due to their low numbers. Participants with evidence of SARS-CoV-2 infection prior to their booster vaccination were excluded in order to examine vaccine and not natural infection-related immunity. Previous SARS-CoV-2 infection was defined as: (i) a positive self-reported polymerase chain reaction (PCR) or lateral flow test (LFT) test; (ii) a positive PCR or LFT test from data linkage; (iii) participants who were seropositive to SARS-CoV-2 anti-nucleocapsid (anti-N) antibodies collected through venous sampling; or (iv) the presence of the Spike antibody prior to December 2020; as these were likely due to natural infection or participation in a vaccination trial.

### Data sources and linkage

For SARS-CoV-2 infections, the primary source of data was the Virus Watch dataset linked to the Second-Generation Surveillance System (SGSS), and contained SARS-CoV-2 test results from hospitalizations (Pillar 1) and community testing (Pillar 2). Linkage was conducted by NHS Digital with the linkage variables being sent in March 2021. The linkage period for SGSS encompassed data from March 2020 until December 2021. Of the 58 566 participants in Virus Watch, 54.7% (*n* = 32 079) participants contained at least one link to SGSS. By January 2022, Virus Watch participants self-reported 326 994 PCR/LFT results; linkage to SGSS provided an extra 10 494 positive events not recorded in Virus Watch.

For vaccination data, the primary source of data was the Virus Watch dataset linked to the National Immunisation Management Service (NIMS) and encompassed vaccinations between October 2020 and December 2021. Of the 58 566 participants in Virus Watch, 63.41% (*n* = 37 138) participants contained at least one link to NIMS. For the booster dose, Virus Watch captured a total of 17 943 vaccinations and NIMS contributed an extra 14 009 vaccinations not recorded in Virus Watch.

### Exposure variables

The exposure variable was the vaccination type (ChAdOx1 or BNT162b2) of the primary vaccine course. Vaccination data in Virus Watch were combined from self-reported and linked data from the NIMS dataset.

In the 11 January 2021 and 18 January 2021 Virus Watch questionnaires, participants were asked about their vaccination status retrospectively. From 25 January 2021 onwards, participants were asked weekly for their vaccination status. Recorded vaccinations from NIMS covered the period 09 October 2020 until 23 December 2021.

### Outcome variables

The primary outcome was SARS-CoV-2 infection using: (i) a positive self-reported PCR or LFT test; or (ii) a positive PCR or LFT test from the linked SGSS data. As we did not link our infection data to symptom data, and our outcome may therefore include asymptomatic cases, we refer to our primary outcome as SARS-CoV-2 infection rather than COVID-19 disease for the purposes of this analysis, although as most testing is undertaken in response to symptoms, the cases will largely represent symptomatic rather than asymptomatic infection.

### Covariates

Self-reported demographic data included age, sex and ethnicity. We included clinically vulnerable status which was derived from self-reported data on immunosuppressive therapy, cancer diagnoses and chronic disease status. Index of multiple deprivation (IMD) quintiles were derived based upon Lower Layer Super Output Areas postcodes submitted during registration, with IMD 5 the least deprived group and IMD 1 the most deprived. Due to small sample sizes, we could not evaluate geographical region or ethnicity in detail; therefore, we classified ethnicity as ‘White British’ or ‘Ethnic Minority’.

### Bias

To estimate the risk of SARS-CoV-2 infection after receiving the booster COVID-19 vaccine, time-to-event analyses could be conducted to estimate the hazard ratio of the incidence of infection since the booster dose. However, evaluating time to SARS-CoV-2 infection may be confounded by the UK’s strategy to prioritize booster vaccinations, based upon age, clinical vulnerability and exposure to the virus (for example, front-line health care workers). On 14 September 2021, the Joint Committee on Vaccination and Immunisation (JCVI) advised that the following groups of people would be offered a third-dose COVID-19 booster vaccine: those living in residential care homes for older adults, all adults aged 50 years or over, front line health and social care workers, all those aged 16 to 49 years with underlying health conditions that put them at higher risk of severe COVID-19 (as set out in the Green book),^12^ adult carers and adult household contacts (aged 16 or over) of immunosuppressed individuals.^13^ This guidance was then updated with the emergence of the Omicron variant in November 2021, so that booster vaccines were offered to all individuals aged 18 to 39 from that point onwards.^14^ Therefore, we believe time-varying confounding by indication existed due to the JCVI’s change in policy to reflect changes in SARs-CoV-2 infection rates driven by the B.1.1.529 variant.

We used methods developed by Hernán and Robins which aim to tackle confounding-by-indication to appropriately estimate the average treatment effect.^10^^,^^15^ This approach includes three primary components: (i) excluding prevalent users of an intervention to estimate the impact of treatment initiation and wash out the effect of previous treatment(s); (ii) use of an intention-to-treat analysis as this is the common estimand in randomized controlled trials; and (iii) use of multiple staggered cohorts to appropriately account for ‘time zero’ (or the start of follow-up since the booster dose). In this study design we consider intention-to-treat to be a complete primary vaccination course followed by an mRNA booster.

To apply Hernán *et al.*’s recommendations, this study: (i) uses eligibility criteria that exclude those who are likely to have protection from SARs-COV-2 (e.g. through prior natural infection): (ii) assigns individuals from the first vaccination dose and disregards changes in course; and (iii) staggers a single cohort based upon booster vaccination date to increase the homogeneity in terms of the eligibility criteria for receiving a booster vaccination at that point in time.

### Statistical analysis

Pooled Cox proportional hazard models were used to estimate the time from booster vaccination until the primary outcome of SARS-CoV-2 infection, loss to follow-up (latest week of reporting to Virus Watch) or end of study (23 January 2022), whichever was earliest. Cohorts were split based upon the date of their booster vaccination, with the cohort dates defined in Table 1. Eight adult cohorts were created by splitting individuals into 14-day intervals based upon booster vaccinations received between 16 September 2021 (the introduction of booster vaccines for the clinically vulnerable or highly exposed^12^) to 05 January 2022 and were followed until 23 January 2022; 14-day intervals were chosen as a balance between providing sufficient participants in each staggered cohort while also trying to account for homogeneity in terms of the eligibility criteria for receiving a booster vaccination and for changes in vaccine policy, such as the announcement of boosters for all by the UK Prime Minister on 12 December 2021.^16^

Multivariable adjustment was conducted using the following variables: age, sex, ethnic minority status, index of multiple deprivation quintile and clinical vulnerability status (clinically vulnerable, clinically extremely vulnerable or none identified). The models from the eight cohorts were pooled using a random-effects meta-analysis. Full case analysis was conducted for all analyses as there was a small amount of missingness (Supplementary Figure S1, available as Supplementary data at *IJE* online). Statistical analysis was conducted using R version 4.0.3.

### Sensitivity analyses

We conducted five sensitivity analyses. First, to examine the effects of prior infection, we included those with a SARSs-CoV-2 infection prior to receiving a booster dose. Second, to examine the effect of including those boosted with mRNA-1273, who were more likely to have received a ChAdOx1 primary course, we only included individuals who were boosted with BNT162b2. Third, to examine the effect of loss to follow-up when using self-reported participant data, we conducted an analysis using only linked data. Fourth, to allow time for the development of antibodies, we started follow-up after 7 days following vaccination. Finally, we conducted an alternative analytical approach where we used matching to condition on vaccination date. The time period used for all sensitivity analyses was the same as for the main analysis.

## Results

Across the eight cohorts, among those who met the eligibility criteria (adults, with a recording of ChAdOx1 or BNT162b2 as a primary course without prior infection or missing data—see Supplementary Figure S1 for more details of the Consolidated Standards of Reporting Trials participant flow chart), a total of 19 159 participants received their booster vaccination between 16 September 2021 to 05 January 2022, of which 4951 participants were included in the laboratory sub-cohort with nasopharyngeal swab and blood collection via venepuncture or finger-prick sampling.

**Figure 1:**
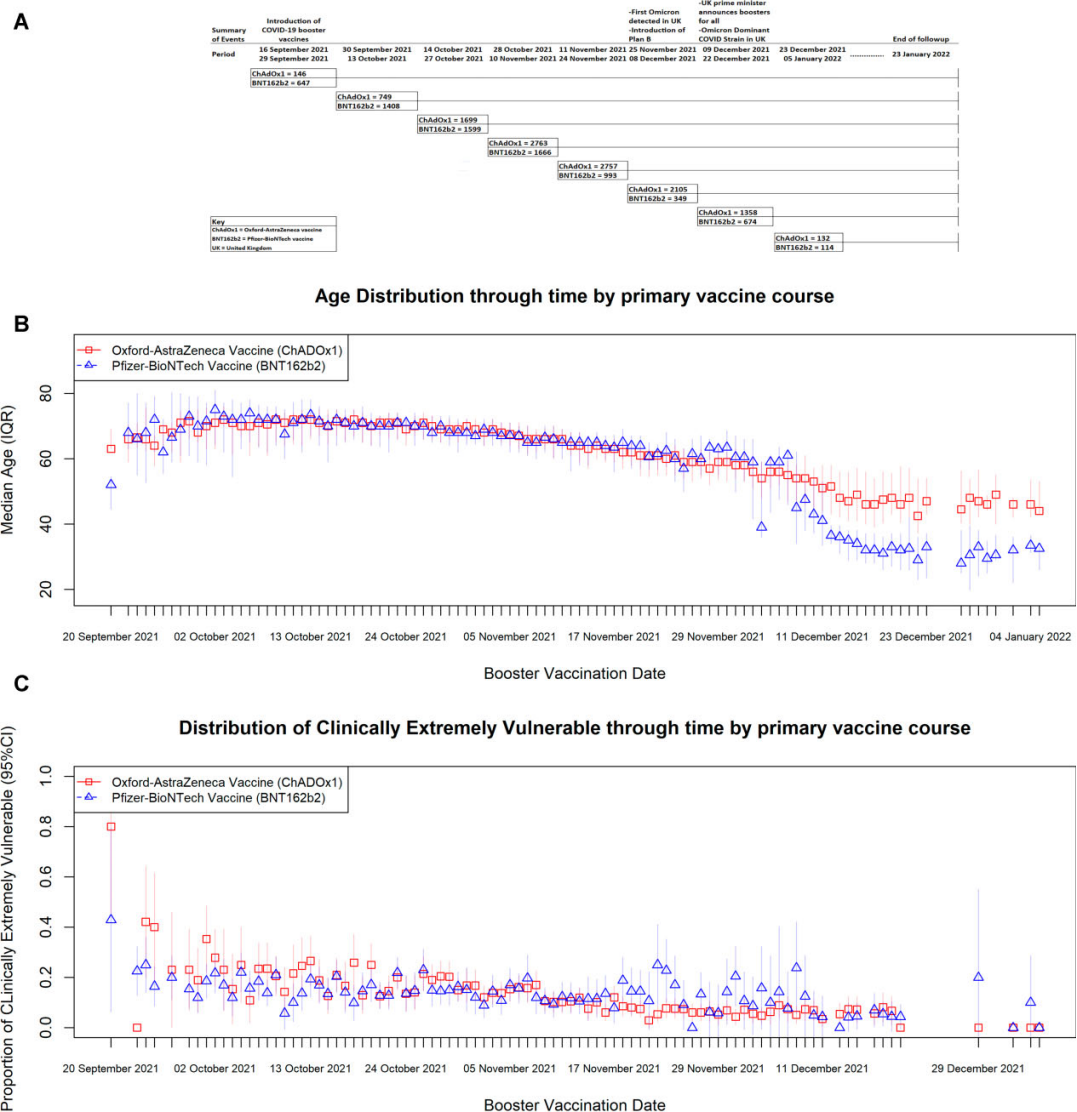
These figures depict the time-varying nature of the distribution of vaccines through time. 1a: Cohort recruitment diagram through time, including a description of time-varying events, particularly the introduction of the booster shot and how the eligibility criteria changed through time to reflect that Omicron (B.1.1.529) transitioned into becoming the dominant variant in England and Wales. The major events included: the introduction of the booster vaccine on 16 September 2021, the initial detection of Omicron (B.1.1.529) in the UK on 27 November 2021, the introduction of Plan B on 8 December 2021, the announcement of boosters for all by the UK Prime minister on 12 December 2021 and the transition of B.1.1.529 into becoming the dominant variant in England and Wales on 17 December 2021. 1b: Distribution of age at time of booster vaccination between 16 September 2021 and 5 January 2022 by primary vaccination course. The gradient for the Oxford–AstraZeneca COVID-19 vaccine (ChAdOx1) was a decrease of 0.27 years per day with a 95% confidence interval (CI) of -0.29 to -0.24, whereas the gradient for the Pfizer–BioNTech COVID-19 vaccine (BNT162b2) was a decrease of 0.41 years per day (95% CI: -0.46 to -0.35). 1c: Distribution of Clinically Extremely Vulnerable status between 16 September 2021 and 5 January 2022 by primary vaccination course. The gradient for ChAdOx1 was a daily decrease of 0.003 proportions of Clinically Extremely Vulnerable status (95% CI: -0.003 to -0.002), whereas the gradient for BNT162b2 was a daily decrease of 0.001 proportions of Clinically Extremely Vulnerable status (95% CI: -0.001 to -0.001)

Compared with Office of National Statistics Mid-2019 population data for England and Wales, participants in Virus Watch were more likely to be female, over 65, live in the southeast of England and live in less deprived areas compared with the general population, and those eligible for this trial were more likely to be White British (Supplementary Table S1). The largest recruitment period was between 28 October 2021 and 10 November 2021, with 4429 participants recruited. The smallest recruitment period was between 23 December 2021 and 05 January 2022, with 246 participants; see Figure 1a for the recruitment timeline of the emulated trials.

Across all eight cohorts, 11 709 individuals had received the ChAdOx1 vaccine as their primary course and 7450 received the BNT162b2 vaccine as their primary course; 83% of the primary exposure was reported to Virus Watch and 13% were obtained through linkage (see Supplementary Figure S2, available as Supplementary data at *IJE* online, for linkage details). Demographic characteristics were broadly similar between ChAdOx1 and BNT162b2 primary course recipients, except for clinical vulnerability status and age, where the BNT162b2 course had slightly more clinically vulnerable patients and an older age group (see Table 2). Due to the staggered cohort design, it is more appropriate to compare individuals who were vaccinated in the same period. In brief, as time advanced, recipients of the booster doses were getting younger; prior to mid-December 2021, the age distributions for both vaccines were similar; however, after this period, those whose primary course was BNT162b2 were younger than their ChAdOx1 counterparts for any given day (Figure 1b). For clinical vulnerability, both ChAdOx1 and BNT162b2 saw a decrease in the daily proportion of those identified as ‘clinically extremely vulnerable’ (Figure 1c); 91% of BNT162b2 (*n* = 6803) primary course individuals received BNT162b2 as their booster dose and 8.7% (*n* = 647) received mRNA-1273 as their booster dose. For individuals who received ChAdOx1 as their primary course, 81% (*n* = 9464) received BNT162b2 as their booster dose and 19% (*n* = 2245) received mRNA-1273 as their booster dose.

**Table 2:** Sociodemographics and clinical breakdown of the cohort stratified by primary vaccination course: Pfizer-BioNTech (BNT162b2) and Oxford-AstraZeneca (ChAdOx1)

Characteristic	*N*	Pfizer (BNT162b2), *N* = 7450^a^	Oxford (ChAdOx1), *N* = 11 709^a^	*P*-value^b^
Age at booster vaccination	19 159	67 (57, 73)	63 (56, 69)	<0.001
Index of Multiple Deprivation	19 159			0.4
quintile				
(Most deprived) 1		531 (7.1%)	888 (7.6%)	
2		1006 (14%)	1607 (14%)	
3		1581 (21%)	2392 (20%)	
4		1923 (26%)	3106 (27%)	
(Least deprived) 5		2409 (32%)	3716 (32%)	
Sex	19 159			0.2
Female		4233 (57%)	6539 (56%)	
Male		3217 (43%)	5170 (44%)	
Ethnicity	19 159			<0.001
Black		29 (0.4%)	41 (0.4%)	
Mixed		66 (0.9%)	90 (0.8%)	
Other Asian		53 (0.7%)	60 (0.5%)	
Other ethnicity		30 (0.4%)	37 (0.3%)	
South Asian		159 (2.1%)	164 (1.4%)	
White British		6658 (89%)	10 701 (91%)	
White Irish		124 (1.7%)	168 (1.4%)	
White other		331 (4.4%)	448 (3.8%)	
Clinically vulnerable status	19 159			<0.001
Clinically extremely vulnerable		1061 (14%)	1373 (12%)	
Clinically vulnerable		2359 (32%)	3360 (29%)	
None identified		4030 (54%)	6976 (60%)	
Booster dose	19 159			<0.001
Moderna		647 (8.7%)	2245 (19%)	
Pfizer		6803 (91%)	9464 (81%)	
Follow-up duration from time of booster dose	19 159	84 (66, 101)	68 (52, 84)	<0.001
SARs-CoV-2^c^ infection	19 159	571 (7.7%)	863 (7.4%)	0.5

a Median (interquartile range); *n* (%); range.

b Wilcoxon rank sum test; Pearson's chi-square test.

c SARs-CoV-2, severe acute respiratory syndrome coronavirus 2.

Both groups were followed (from booster vaccination) up to a maximum of 129 days (from 16 September 2021 until 23 January 2022), with ChAdOx1 individuals producing a median follow-up duration of 68 days with an interquartile range (IQR) between 52 days and 84 days, whereas BNT162b2 individuals had a median follow-up duration of 84 days (IQR: 66, 101). At 129 days, ChAdOx1 participants experienced an incidence of 73.7 infections per 1000 (95% CI: 69.0 to 78.6) vaccinated individuals whereas BNT162b2 participants experienced an incidence of 76.6 infections per 1000 (95% CI: 70.7 to 82.9) vaccinated individuals; see Supplementary Figure S3 (available as Supplementary data at *IJE* online) for Kaplan–Meier plots for all cohorts. In all, 92% of first SARS-CoV-2 infections after the booster dose were self-reported and 8% were obtained through linkage (see Supplementary Figure S2 for linkage details).

### Crude analysis

ChAdOx1 produces a pooled unadjusted HR of 0.97 (95%: 0.86 to 1.09) in the incidence of SARS-CoV-2 infection since the booster dose when compared with BNT162b2 at up to 129 days.

**Figure 2:**
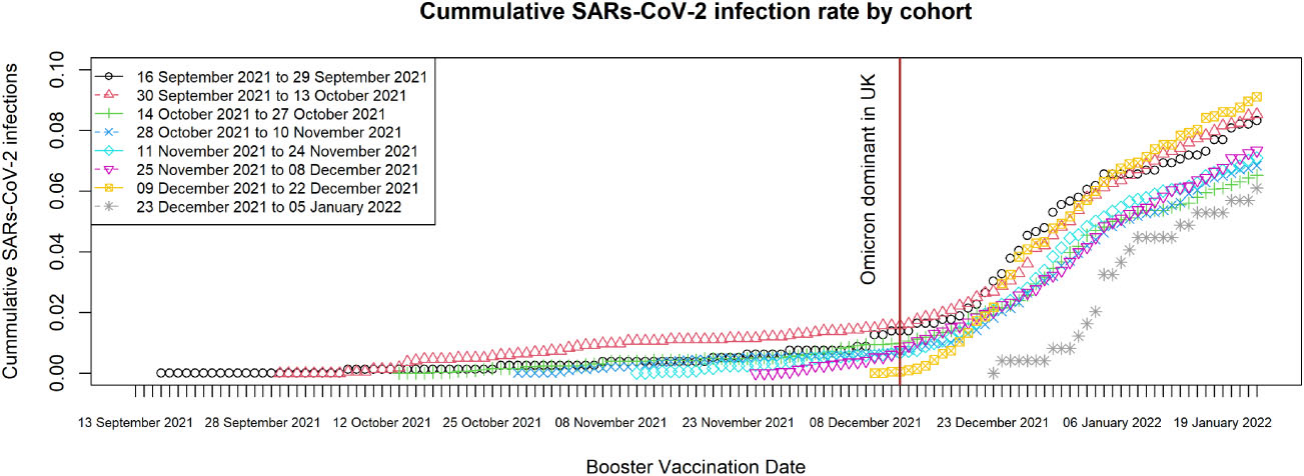
Cumulative SARs-CoV-2 incidence rate by vaccination date cohort. Between the date of booster vaccination and end of study, the rates of increase (% increase per day) in cumulative proportions by cohort were: 16 September to 29 September 2021: 0.059 (95% CI: 0.052 to 0.065); 30 September to 13 October 2021: 0.067 (95% CI: 0.061to- 0.074); 14 October to 27 October 2021: 0.064 (95% CI: 0.057 to 0.071); 28 October to 10 November 2021: 0.079 (95% CI: 0.071 to 0.088); 11 November to 24 November 2021: 0.110 (95% CI: 0.101 to 0.119); 25 November to 08 December 2021: 0.141 (95% CI: 0.133 to 0.148); 09 December to 22 December 2021: 0.237 (95% CI: 0.227 to 0.247); 13 December to 05 January 2022: 0.235 (95% CI: 0.215 to 0.255)

The change in rates of cumulative infections changed for all cohorts since the B.1.1.529 variant became the dominant strain of SARs-CoV-2 in the UK; those who received their booster dose earlier had a longer period of stable cumulative incidence compared with those who received their booster dose closer or after the B.1.1.529 variant was declared the dominant SARs-CoV-2 strain in the UK; see Figure 2 for the cumulative incidence rate of SARs-COV-2 infections grouped by vaccination cohort.

### Adjusted analysis

**Figure 3:**
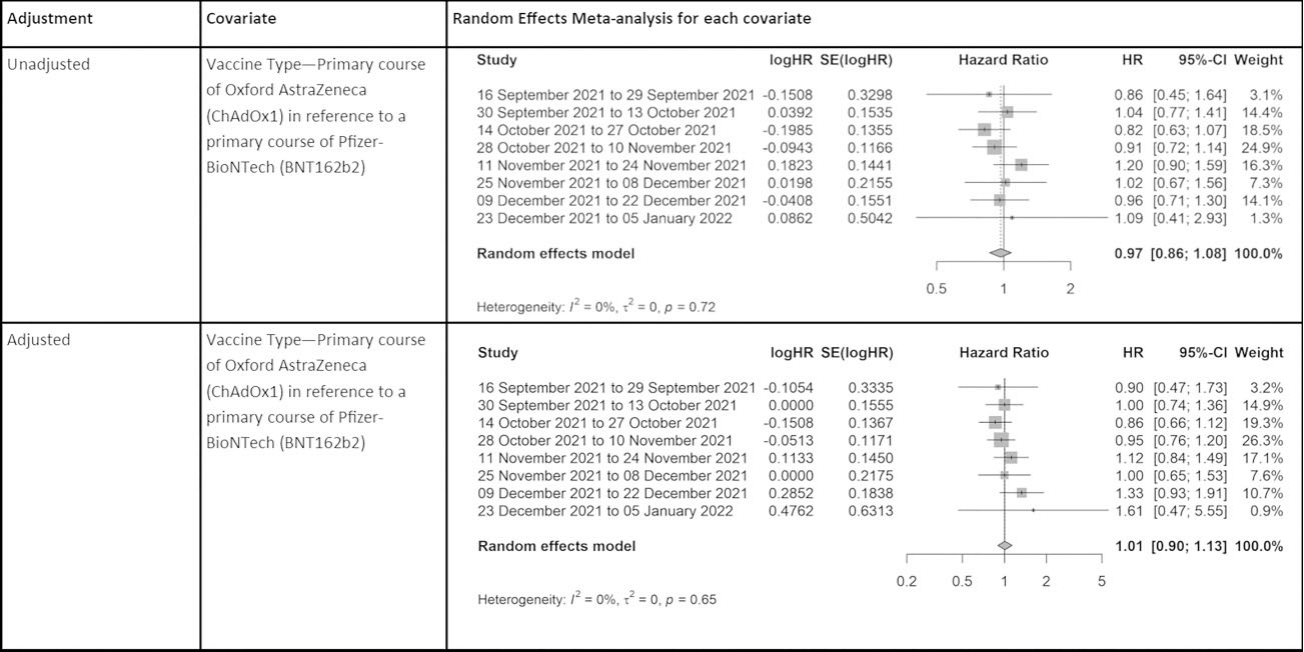
Adjusted and unadjusted random-effects meta-analysis for primary vaccine type only: logHR, natural log of the hazard ratio; SE(logHR), standard error of the hazard ratio; HR, hazard ratio; 95% CI, 95% confidence interval

After adjusting for age at vaccination, clinical vulnerability, IMD quintile, minority ethnic status and sex, the adjusted hazard ratio (aHR) for ChAdOx1 was 1.01 (95% CI: 0.90 to 1.13), suggesting no difference in vaccination effectiveness when compared with BNT162b2 after receiving an mRNA-based booster vaccine (see Figure 3).

### Sensitivity analyses

The first sensitivity analysis included those with a SARS-CoV-2 infection prior to receiving a booster dose. This allows our analysis to be more pragmatic due to the rising SARS-CoV-2 infection rates even in double-vaccinated individuals. We adjusted for prior infection in our modelling to account for the impact of natural infection on the protection provided by the nucleocapsid antibody. This increased our cohort to 21 947 individuals, with 8451 receiving BNT162b2 and 13 496 receiving ChAdOx1 as their respective primary vaccine courses. Sociodemographic characteristics remained similar to the primary analysis (see Supplementary Table S2 and Supplementary Figure S4, available as Supplementary data at *IJE* online). Both the univariable and the multivariable models remained similar to the primary analysis where the aHR for SARS-CoV-2 infection after vaccination was 1.02 (95% CI: 0.91 to 1.14)

The second sensitivity analysis controlled for the booster dose’s manufacturer. In our primary analysis, those who originally received ChAdOx1 as their primary dose were twice as likely to receive mRNA-1273 as a booster dose when compared with those who received BNT162b2 as their primary vaccine course (*p*-value <0.001). To control for this difference in booster dose manufacturer, we conducted a sensitivity analysis that only included individuals who were boosted with BNT162b2. Therefore, our comparisons would include those who have had three courses of BNT162b2 against those who had a primary course of ChAdOx1 with a booster dose of BNT162b2. Under this set of assumptions, the cohort reduced to 16 267 individuals with 6803 individuals receiving BNT162b2 as their primary course and 9464 individuals receiving ChAdOx1 as their primary course. The pooled aHR for a primary dose of ChAdOx1 was 1.06 (95% CI: 0.90 to 1.25) in reference to a primary dose of BNT162b2; see Supplementary Table S3 and Supplementary Figure S5 (available as Supplementary data at *IJE* online) for more details about this sensitivity analysis.

To reduce the impact of drop-out (11% dropped out), which may exclude individuals with more severe outcomes (e.g. death), the third sensitivity analysis was conducted only using participants with SGSS outcome data through successful linkage by NHS Digital; this allowed data to be used after a participant had stopped self-reporting results to Virus Watch. Outcomes for this sensitivity analysis used self-report and linked data but restricted the analysis to a cohort who had linked data during the study period. Follow-up was defined from booster vaccination until first SARs-CoV-2 infection (self-reported or linked), end of data (latest SGSS submitted swab or Virus Watch reporting week, whichever was latest) or end of study (23 January 2022). By using those who were successfully linked by NHS Digital, the cohort reduced to 16 059 individuals with 6252 receiving BNT162b2 as their primary course and 9807 individuals receiving ChAdOx1 as their primary course. The pooled aHR for a primary dose of ChAdOx1 was 0.98 (95% CI: 0.86 to 1.11) in reference to a primary dose of BNT162b2; see Supplementary Table S4 and Supplementary Figure S6 (available as Supplementary data at *IJE* online) for more details about this sensitivity analysis.

Under the fourth sensitivity analysis, we changed our follow-up window from directly after the booster vaccination to the 8th day after vaccination, to allow a 1-week window to account for antibody production. Therefore, those who were infected with SARs-COV-2 between the date of their booster vaccination up to 7 days after were removed from our analysis. This reduced the cohort to 18 976 individuals, with 7371 individuals receiving BNT162b2 as the primary course and 11 605 individuals receiving ChAdOx1 as their primary course. The pooled aHR for a primary dose of ChAdOx1 was 1.01 (95% CI: 0.89 to 1.14) in reference to a primary dose of BNT162b2; see Supplementary Table S5 and Supplementary Figure S7 (available as Supplementary data at *IJE* online) for more details about this sensitivity analysis.

We have also produced the results using matching instead of staggering our cohort by vaccination time period. We matched ChAdOx1 participants to their mRNA counterparts based upon age at booster vaccination, sex, IMD quintile, minority ethnic status and booster vaccination date. Exact matching was used instead of probabilistic matching (such as propensity score matching), as younger clinically vulnerable individuals who were vaccinated earlier could be matched with older less clinically vulnerable individuals as per the JCVI’s prioritization schedule. Under this analysis, our cohort size reduced to 2952 individuals with 1343 individuals receiving BNT162b2 as their primary course and 1609 individuals receiving ChAdOx1 as their primary course. The aHR for a primary dose of ChAdOx1 was 1.01 (95% CI: 0.75 to 1.35) in reference to a primary dose of BNT162b2; see Supplementary Table S6 (available as Supplementary data at *IJE* online) for more details about this analysis.

## Discussion

Our analysis was conducted in a community cohort of 19 159 people across England and Wales who received their booster vaccination between 16 September 2021 and 05 January 2022. We followed people up for risk of SARS-CoV-2 infection between 16 September 2021 and 23 January 2022, and found that people who received ChAdOx1 vaccinations as their primary course had no difference in the incidence of SARS-CoV-2 infection compared with BNT162b2 during follow-up after we accounted for differences in sociodemographic and clinical characteristics between our comparison groups as well as the time of vaccination.

Our analysis used a community sample design from across England and Wales in a cohort with diversity in terms of age, sex and geographical location. We estimated effectiveness in a cohort with a median follow-up of 2 months after a booster vaccination, and the majority of infections occurred during a period when B.1.1.529 became the dominant variant in the UK. A particular strength of our analysis was our ability to estimate vaccine effectiveness in a cohort that included large numbers of people who were either clinical vulnerable or clinically extremely vulnerable—a group that was prioritized for booster doses based upon need. Using this sample, we applied a trial emulation framework to mitigate against confounding by indication. As a result of this study design, our results are more likely to reflect a randomized controlled trial evaluating the same question.

Our staggered cohort approach has additional strengths. First, it enables us to account for the demographic and clinical risk factors of vaccines and make comparisons between similar demographically and clinically similar groups; this was demonstrated in the changing median age and declining clinical vulnerable status of our cohorts. Second, our approach helps control for changes in SARS-CoV-2 transmission rates driven by changes in public health policy such as the vaccination efforts (e.g. prioritized distribution), mask usage and limitations on movement as well the emergence of new SARS-CoV-2 variants and their transition to becoming the dominant SARs-CoV-2 strain in England and Wales, which we graphically demonstrated in Figure 2. Third, our strategy helps us mitigate the impact of varying antibody waning trajectories that arise from the age and clinical vulnerabilities differences of our staggered cohorts, as this allows similar individuals to have similar time to wane. Therefore our approach controls for measured time-varying confounders and, to some extent, it goes some way to mitigating against the impact of unmeasured time-varying confounders (i.e. SARs-CoV-2 strain and waning trajectories). Finally, our sensitivity analyses provide reassurance that our results are not substantially affected by our analytical decisions about the inclusion of people with prior infection, biases in vaccine type received by different demographic groups, biases due to loss to follow-up or the time period used to define the start of follow-up.

Due to the reliance on self-reported observational studies, there is a risk of inconsistent and inaccurate data recording; however, this was mitigated through linkage to external data sources such as SGSS to complement missing incidence SARS-CoV-2 infections and NIMs to complement missing vaccination data. We measured the risk of SARS-CoV-2 infection as our primary outcome, and as this precedes hospitalization or death, we were not able to look at these more severe outcomes, which is a limitation of our study. Changes in the risk of severe infection would, in the context of Virus Watch, lead to biases in the comparison of vaccine effectiveness. Our staggered cohort design, which creates comparison groups with similar risk factors, should reduce this bias, in addition to our use of linkage to external data sources such as SGSS, which aims to reduce loss to follow-up for those with severe outcomes unable to self-report results to Virus Waych. Our use of observational data may mean that there is residual and uncontrolled confounding. Unlike test-negative designs, our approach does not implicitly control for differences in testing behaviour between groups, but since we are comparing vaccine regimens rather than vaccinated and un-vaccinated individuals, we do not expect confounding by differential testing behaviour. Using multiple staggered cohorts reduces each cohort size, and as a result we had difficulties with analysing certain covariates such as geographical region and ethnicity, which we had to combine into an aggregated category. We did not include occupation or geographical risk in our analyses, and these may result in imbalances in the comparison arms, as both risks of exposure to SARS-CoV-2 infection and access to BNT162b2 varied geographically (due to its cold storage requirements) and by occupation (e.g. health and social care workers). We were also unable to stratify based upon covariates such as age, due to the age-based roll-out of the booster vaccine based upon the JCVI’s change of eligibility through time. Stratification by age was also difficult due to the differential time without the risk of the B.1.1.529 experienced by different age groups (see Supplementary Figure S8, available as Supplementary data at *IJE* online). Participants included in the current trial are older than those in the wider Virus Watch cohort and target population. Vaccine-induced immunity is lower in older adults and therefore our absolute estimates of risk may be lower than those found in the target population. We found that after mid-December 2021, the age distributions for those whose primary course was BNT162b2 were younger than their ChAdOx1 counterparts for any given day, and as a result our findings may under-estimate the protective effect of ChAdOx1 compared with BNT162b2.

## Conclusion

We found evidence of the same effectiveness of a primary course of BNT162b2 compared with a primary course of ChAdOx1 vaccines against SARS-CoV-2 infection after receiving a booster vaccination in England and Wales, a finding that contrasts with previous analysis showing that prior to such boosters, those who had ChAdOx1 as their primary course were at higher risk of a SARs-CoV-2 infection.^2^ In other analyses we have demonstrated that antibody levels are substantially higher following a primary course of BNT162b2 than following a primary course of ChAdOx1, that antibodies wane following a log-linear pattern and that risk of infection is increased in those with lower antibody levels.^3^ Thus, we hypothesize that differential effectiveness of BNT162b2 and ChadOx1 primary courses against infection are related to different antibody levels. We have also shown that following an mRNA booster dose, antibody levels are similar regardless of the primary course, and we hypothesize that this accounts for the similar effectiveness of mRNA boosters regardless of primary regimen.^17^ Our findings demonstrate the importance of mRNA booster doses in maintaining protection, particularly for those with a primary course of ChadOx1.

## Ethics approval

This study has been approved by the Hampstead NHS Health Research Authority Ethics Committee, ethics approval number 20/HRA/2320.

## Supplementary Material

dyad002_Supplementary_Data

## Data Availability

We aim to share aggregate data from this project on our website and via a ‘Findings so far’ section on our website [https://ucl-virus-watch.net/]. We also share some individual record-level data on the Office of National Statistics Secure Research Service (DOI: 10.57906/s5f5-nq13). In sharing the data, we will work within the principles set out in the UKRI Guidance on best practice in the management of research data. Access to use of the data while research is being conducted will be managed by the Chief Investigators (A.C.H. and R.W.A.) in accordance with the principles set out in the UKRI guidance on best practice in the management of research data. We will put analysis code on publicly available repositories to enable their reuse.
