# Supplementary material for: Comparative effectiveness of different primary vaccination courses on mRNA-based booster vaccines against SARs-COV-2 infections: a time-varying cohort analysis using trial emulation in the Virus Watch community cohort
Source: Int J Epidemiol. 2023 Jan 19;52(2):342–54. doi: 10.1093/ije/dyad002 (PMC10114109; doi:10.1093/ije/dyad002)

Supplementary Table S1: Comparison of the Virus Watch Cohort and the Office of National Statistics (ONS) breakdown of England and Wales

| Characteristic | All Virus Watch Participants on 05 February 2021  (n = 58,566) | Virus Watch Participants eligible for this trial (n = 19,159) | ONS (%)* |
| --- | --- | --- | --- |
| **Age Group** | | | |
| 0-15 | 7,364 (13%) | - | 19.1% |
| 16-24 | 3,492 (6.0%) | 220 (1.1%) | 10.6% |
| 25-44 | 11,713 (20%) | 1,797 (9.4%) | 26.1% |
| 45-64 | 19,631 (34%) | 7,557 (39%) | 25.6% |
| 65+ | 16,365 (28%) | 9,585 (50%) | 18.5% |
| **Ethnicity** | | | |
| White British | 40,436 (69%) | 17,359 (91%) | 80.5% |
| White Irish | 670 (1.1%) | 292 (1.5%) | 0.9% |
| White Other | 2,815 (4.8%) | 779 (4.1%) | 4.4% |
| Mixed | 996 (1.7%) | 156 (0.8%) | 2.2% |
| South Asian | 2,726 (4.7%) | 323 (1.7%) | 5.3% |
| Other Asian | 427 (0.7%) | 113 (0.6%) | 2.2% |
| Black | 491 (0.8%) | 70 (0.4%) | 3.3% |
| Other Ethnicity | 287 (0.5%) | 67 (0.3%) | 1% |
| Prefer not to say | 192 (0.3%) | - | - |
| Missing | 9,526 (16%) | - |  |
| **Sex** | | | |
| Male | 21,663 (37%) | 8,387 (44%) | 49.4% |
| Female | 27,476 (47%) | 10,772 (56%) | 50.6% |
| Missing/Suppressed | 9,427 (16%) |  | - |
| **Region** | | | |
| East Midlands | 4,936 (8.4%) | 1,821 (9.5%) | 4.5% |
| East of England | 10,535 (18%) | 4,200 (22%) | 12.4% |
| London | 9,079 (16%) | 2,029 (11%) | 9.3% |
| North East | 2,524 (4.3%) | 949 (5.0%) | 8.1% |
| North West | 5,565 (9.5%) | 2,041 (11%) | 10% |
| South East | 9,833 (17%) | 3,784 (20%) | 10.5% |
| South West | 3,952 (6.7%) | 1,648 (8.6%) | 10.1% |
| Wales | 1,528 (2.6%) | 453 (2.4%) | 15.1% |
| West Midlands | 3,014 (5.1%) | 1,146 (6.0%) | 9.5% |
| Yorkshire and the Humber | 3,033 (5.2%) | 1,060 (5.5%) | 5.3% |
| Missing | 4,567 (7.8%) | 28 | - |
| **Index of Multiple Deprivation (Quintiles)** | | | |
| (Most Deprived) 1 | 5,946 (11%) | 1,419 (7.4%) | 20% |
| 2 | 9,158 (17%) | 2,613 (14%) | 20% |
| 3 | 10,788 (20%) | 3,973 (21%) | 20% |
| 4 | 13,035 (24%) | 5,029 (26%) | 20% |
| (Least Deprived) 5 | 15,062 (28%) | 6,125(32%) | 20% |

*ONS data for age and region drawn from Mid-2019 [Estimates of the Population for the UK, England, and Wales, Scotland and Northern Ireland](https://www.ons.gov.uk/peoplepopulationandcommunity/populationandmigration/populationestimates/datasets/populationestimatesforukenglandandwalesscotlandandnorthernireland) (figures for England and Wales).

Supplementary Table S2:  Demographics breakdown of the sensitivity analysis one where those with a prior severe acute respiratory syndrome coronavirus 2 (SARs-CoV-2) infection were not excluded. The table is stratified by primary vaccination course: Pfizer-BioNTech (BNT162b2) and Oxford-AstraZeneca (ChAdOx1)

| **Characteristic** | **N** | **Pfizer**  **(BNT162b2),**  **N = 8,451^1^** | **Oxford (ChAdOx1),**  **N = 13,496^1^** | **p-value^2^** |
| --- | --- | --- | --- | --- |
| **Age at booster** | 21,947 | 66 (55, 72) | 63 (55, 69) | <0.001 |
| **Index of Multiple Deprivation**  **Quintile** | 21,947 |  |  | 0.7 |
| (Least Deprived) 1 |  | 639 (7.6%) | 1,031 (7.6%) |  |
| 2 |  | 1,189 (14%) | 1,870 (14%) |  |
| 3 |  | 1,773 (21%) | 2,741 (20%) |  |
| 4 |  | 2,186 (26%) | 3,587 (27%) |  |
| (Most Deprived) 5 |  | 2,664 (32%) | 4,267 (32%) |  |
| **Sex** | 21,947 |  |  | 0.047 |
| Female |  | 4,842 (57%) | 7,548 (56%) |  |
| Male |  | 3,609 (43%) | 5,948 (44%) |  |
| **Ethnicity** | 21,947 |  |  | <0.001 |
| Black |  | 33 (0.4%) | 44 (0.3%) |  |
| Mixed |  | 83 (1.0%) | 108 (0.8%) |  |
| Other Asian |  | 64 (0.8%) | 69 (0.5%) |  |
| Other Ethnicity |  | 34 (0.4%) | 38 (0.3%) |  |
| South Asian |  | 187 (2.2%) | 208 (1.5%) |  |
| White British |  | 7,536 (89%) | 12,323 (91%) |  |
| White Irish |  | 136 (1.6%) | 188 (1.4%) |  |
| White Other |  | 378 (4.5%) | 518 (3.8%) |  |
| **Clinical Vulnerability** | 21,947 |  |  | <0.001 |
| Clinically extremely vulnerable |  | 1,205 (14%) | 1,549 (11%) |  |
| Clinically vulnerable |  | 2,666 (32%) | 3,879 (29%) |  |
| None Identified |  | 4,580 (54%) | 8,068 (60%) |  |
| **Booster Vaccination Manufacture** | 21,947 |  |  | <0.001 |
| Moderna - mRNA-1273 |  | 779 (9.2%) | 2,753 (20%) |  |
| Pfizer - BNT162b2 |  | 7,672 (91%) | 10,743 (80%) |  |
| **Follow-up duration since booster vaccination** | 21,947 | 75 (56, 91) | 58 (41, 73) | <0.001 |
| **SARs-CoV-2^3^ infection (Before booster)** | 21,947 | 1,001 (12%) | 1,787 (13%) | 0.003 |
| **SARs-CoV-2^3^ infection (after booster)** | 21,947 | 587 (6.9%) | 884 (6.6%) | 0.3 |
| ^1^ Median (Interquartile range); n (%); Range  ^2^ Wilcoxon rank sum test; Pearson's Chi-squared test  ^3^ SARs-CoV-2 = severe acute respiratory syndrome coronavirus 2 | | | | |

Supplementary Table S3: Demographic breakdown for sensitivity analysis two, where vaccination history was restricted to those who received either a primary course of the Pfizer-BioNTech vaccine (BNT162b2), with a booster course of BNT162b2 against those who received a primary dose of the Oxford-AstraZeneca vaccine (ChAdOx1) with a booster dose of BNT162b2.

| **Characteristic** | **N** | **Pfizer (BNT162b2),**  **N = 6,803^1^** | **Oxford (ChAdOx1),**  **N = 9,464^1^** | **p-value^2^** |
| --- | --- | --- | --- | --- |
| **Age at booster vaccination** | 16,267 | 67 (59, 73) | 65 (57, 70) | <0.001 |
| **Index of Multiple Deprivation**  **Quintile** | 16,267 |  |  | 0.4 |
| (Least Deprived) 1 |  | 470 (6.9%) | 695 (7.3%) |  |
| 2 |  | 912 (13%) | 1,287 (14%) |  |
| 3 |  | 1,450 (21%) | 1,934 (20%) |  |
| 4 |  | 1,758 (26%) | 2,532 (27%) |  |
| (Most Deprived) 5 |  | 2,213 (33%) | 3,016 (32%) |  |
| **Sex** | 16,267 |  |  | 0.3 |
| Female |  | 3,871 (57%) | 5,303 (56%) |  |
| Male |  | 2,932 (43%) | 4,161 (44%) |  |
| **Ethnicity** | 16,267 |  |  | 0.002 |
| Black |  | 29 (0.4%) | 37 (0.4%) |  |
| Mixed |  | 55 (0.8%) | 71 (0.8%) |  |
| Other Asian |  | 44 (0.6%) | 42 (0.4%) |  |
| Other Ethnicity |  | 27 (0.4%) | 28 (0.3%) |  |
| South Asian |  | 143 (2.1%) | 136 (1.4%) |  |
| White British |  | 6,102 (90%) | 8,672 (92%) |  |
| White Irish |  | 121 (1.8%) | 131 (1.4%) |  |
| White Other |  | 282 (4.1%) | 347 (3.7%) |  |
| **Clinical Vulnerability** | 16,267 |  |  | <0.001 |
| Clinically extremely vulnerable |  | 1,003 (15%) | 1,236 (13%) |  |
| Clinically vulnerable |  | 2,168 (32%) | 2,803 (30%) |  |
| None Identified |  | 3,632 (53%) | 5,425 (57%) |  |
| **Follow-up duration since booster vaccination** | 16,267 | 87 (71, 102) | 74 (57, 87) | <0.001 |
| **SARs-CoV-2^3^ infection (after booster)** | 16,267 | 517 (7.6%) | 708 (7.5%) | 0.8 |
| ^1^ Median (Interquartile range); n (%); Range  ^2^ Wilcoxon rank sum test; Pearson's Chi-squared test  ^3^ SARs-CoV-2 = severe acute respiratory syndrome coronavirus 2 | | | | |

Supplementary Table S4: Demographic breakdown for sensitivity analysis three, where participants with linkage were analysed to improve the dropout rate. The table is stratified by primary vaccination course: Pfizer-BioNTech (BNT162b2) and Oxford-AstraZeneca (ChAdOx1)

| **Characteristic** | **N** | **Pfizer (BNT162b2),**  **N = 6,252^1^** | **Oxford (ChAdOx1),**  **N = 9,807^1^** | **p-value^2^** |
| --- | --- | --- | --- | --- |
| **Age at booster** | 16,059 | 67 (57, 73) | 63 (56, 69) | <0.001 |
| **Index of Multiple Deprivation**  **Quintile** | 16,059 |  |  | 0.12 |
| (Least Deprived) 1 |  | 422 (6.7%) | 747 (7.6%) |  |
| 2 |  | 841 (13%) | 1,358 (14%) |  |
| 3 |  | 1,336 (21%) | 2,050 (21%) |  |
| 4 |  | 1,630 (26%) | 2,609 (27%) |  |
| (Most Deprived) 5 |  | 2,023 (32%) | 3,043 (31%) |  |
| **Sex** | 16,059 |  |  | 0.9 |
| Female |  | 3,489 (56%) | 5,460 (56%) |  |
| Male |  | 2,763 (44%) | 4,347 (44%) |  |
| **Ethnicity** | 16,059 |  |  | 0.027 |
| Black |  | 22 (0.4%) | 31 (0.3%) |  |
| Mixed |  | 50 (0.8%) | 75 (0.8%) |  |
| Other Asian |  | 45 (0.7%) | 55 (0.6%) |  |
| Other Ethnicity |  | 26 (0.4%) | 35 (0.4%) |  |
| South Asian |  | 143 (2.3%) | 158 (1.6%) |  |
| White British |  | 5,585 (89%) | 8,922 (91%) |  |
| White Irish |  | 104 (1.7%) | 150 (1.5%) |  |
| White Other |  | 277 (4.4%) | 381 (3.9%) |  |
| **Clinical Vulnerability** | 16,059 |  |  | <0.001 |
| Clinically extremely vulnerable |  | 974 (16%) | 1,259 (13%) |  |
| Clinically vulnerable |  | 1,927 (31%) | 2,742 (28%) |  |
| None Identified |  | 3,351 (54%) | 5,806 (59%) |  |
| **Follow-up duration since booster vaccination** | 16,059 | 83 (66, 100) | 68 (52, 84) | <0.001 |
| **SARs-CoV-2 infection^3^ (after booster)** | 16,059 | 490 (7.8%) | 729 (7.4%) | 0.3 |
| ^1^ Median (Interquartile range); n (%); Range  ^2^ Wilcoxon rank sum test; Pearson's Chi-squared test  ^3^ SARs-CoV-2 = severe acute respiratory syndrome coronavirus 2 | | | | |

Supplementary Table S5: Demographic breakdown for sensitivity analysis four, the start of follow-up was 8 days after the recorded booster vaccination date to account for the time it takes for antibody development. The table is stratified by primary vaccination course: Pfizer-BioNTech (BNT162b2) and Oxford-AstraZeneca (ChAdOx1)

| **Characteristic** | **N** | **Pfizer (BNT162b2),**  **N = 7,371^1^** | **Oxford (ChAdOx1),**  **N = 11,605^1^** | **p-value^2^** |
| --- | --- | --- | --- | --- |
| **Age at booster** | 18,976 | 67 (57, 73) | 63 (56, 69) | <0.001 |
| **Index of Multiple Deprivation**  **Quintile** | 18,976 |  |  | 0.3 |
| (Least Deprived) 1 |  | 522 (7.1%) | 879 (7.6%) |  |
| 2 |  | 997 (14%) | 1,587 (14%) |  |
| 3 |  | 1,565 (21%) | 2,370 (20%) |  |
| 4 |  | 1,901 (26%) | 3,083 (27%) |  |
| (Most Deprived) 5 |  | 2,386 (32%) | 3,686 (32%) |  |
| **Sex** | 18,976 |  |  | 0.2 |
| Female |  | 4,185 (57%) | 6,478 (56%) |  |
| Male |  | 3,186 (43%) | 5,127 (44%) |  |
| **Ethnicity** | 18,976 |  |  | <0.001 |
| Black |  | 28 (0.4%) | 39 (0.3%) |  |
| Mixed |  | 62 (0.8%) | 89 (0.8%) |  |
| Other Asian |  | 51 (0.7%) | 59 (0.5%) |  |
| Other Ethnicity |  | 30 (0.4%) | 36 (0.3%) |  |
| South Asian |  | 154 (2.1%) | 158 (1.4%) |  |
| White British |  | 6,600 (90%) | 10,617 (91%) |  |
| White Irish |  | 122 (1.7%) | 164 (1.4%) |  |
| White Other |  | 324 (4.4%) | 443 (3.8%) |  |
| **Clinical Vulnerability** | 18,976 |  |  | <0.001 |
| Clinically extremely vulnerable |  | 1,053 (14%) | 1,363 (12%) |  |
| Clinically vulnerable |  | 2,335 (32%) | 3,334 (29%) |  |
| None Identified |  | 3,983 (54%) | 6,908 (60%) |  |
| **Follow-up duration since booster vaccination** | 18,976 | 85 (67, 101) | 69 (52, 84) | <0.001 |
| **SARs-CoV-2 infection^3^ (after booster)** | 18,976 | 530 (7.2%) | 799 (6.9%) | 0.4 |
| ^1^ Median (Interquartile range); n (%); Range  ^2^ Wilcoxon rank sum test; Pearson's Chi-squared test  ^3^ SARs-CoV-2 = severe acute respiratory syndrome coronavirus 2 | | | | |

Supplementary Table S6: Demographic breakdown for sensitivity analysis five, where we conducted matching on booster vaccination date, age, Index of Multiple Deprivation quintile, sex, minority ethnic status and clinical vulnerability status. The table is stratified by primary vaccination course: Pfizer-BioNTech (BNT162b2) and Oxford-AstraZeneca (ChAdOx1)

| **Characteristic** | **N** | **Pfizer (BNT162b2),**  **N = 1,343^1^** | **Oxford (ChAdOx1),**  **N = 1,609^1^** | **p-value^2^** |
| --- | --- | --- | --- | --- |
| **Age at booster** | 2,952 | 68 (65, 72) | 68 (65, 72) | 0.2 |
| **Index of Multiple Deprivation**  **Quintile** | 2,952 |  |  | 0.7 |
| (Least Deprived) 1 |  | 23 (1.7%) | 25 (1.6%) |  |
| 2 |  | 91 (6.8%) | 100 (6.2%) |  |
| 3 |  | 245 (18%) | 273 (17%) |  |
| 4 |  | 389 (29%) | 458 (28%) |  |
| (Most Deprived) 5 |  | 595 (44%) | 753 (47%) |  |
| **Sex** | 2,952 |  |  | 0.7 |
| Female |  | 766 (57%) | 929 (58%) |  |
| Male |  | 577 (43%) | 680 (42%) |  |
| **Minority Ethnic Status** | 2,952 |  |  | 0.8 |
| White British |  | 1,330 (99%) | 1,595 (99%) |  |
| Minority ethnic |  | 13 (1.0%) | 14 (0.9%) |  |
| **Clinical Vulnerability** | 2,952 |  |  | 0.063 |
| Clinically extremely vulnerable |  | 888 (66%) | 1,129 (70%) |  |
| Clinically vulnerable |  | 74 (5.5%) | 78 (4.8%) |  |
| None Identified |  | 381 (28%) | 402 (25%) |  |
| **Follow-up duration since booster vaccination** | 2,952 | 79 (70, 89) | 78 (69, 88) | 0.085 |
| **SARs-CoV-2 infection^3^ (after booster)** | 2,952 | 85 (6.3%) | 101 (6.3%) | >0.9 |
| ^1^ Median (Interquartile range); n (%); Range  ^2^ Wilcoxon rank sum test; Pearson's Chi-squared test  ^3^ SARs-CoV-2 = severe acute respiratory syndrome coronavirus 2 | | | | |

Supplementary Figure S1: Consolidated Standards of Reporting Trials (CONSORT) diagram for how the cohort was derived. In the following diagram, mRNA is abbreviated for messenger ribonucleic acid, SARs-CoV-2 is abbreviated for severe acute respiratory syndrome coronavirus 2, and IMD is abbreviated for index of multiple deprivation.


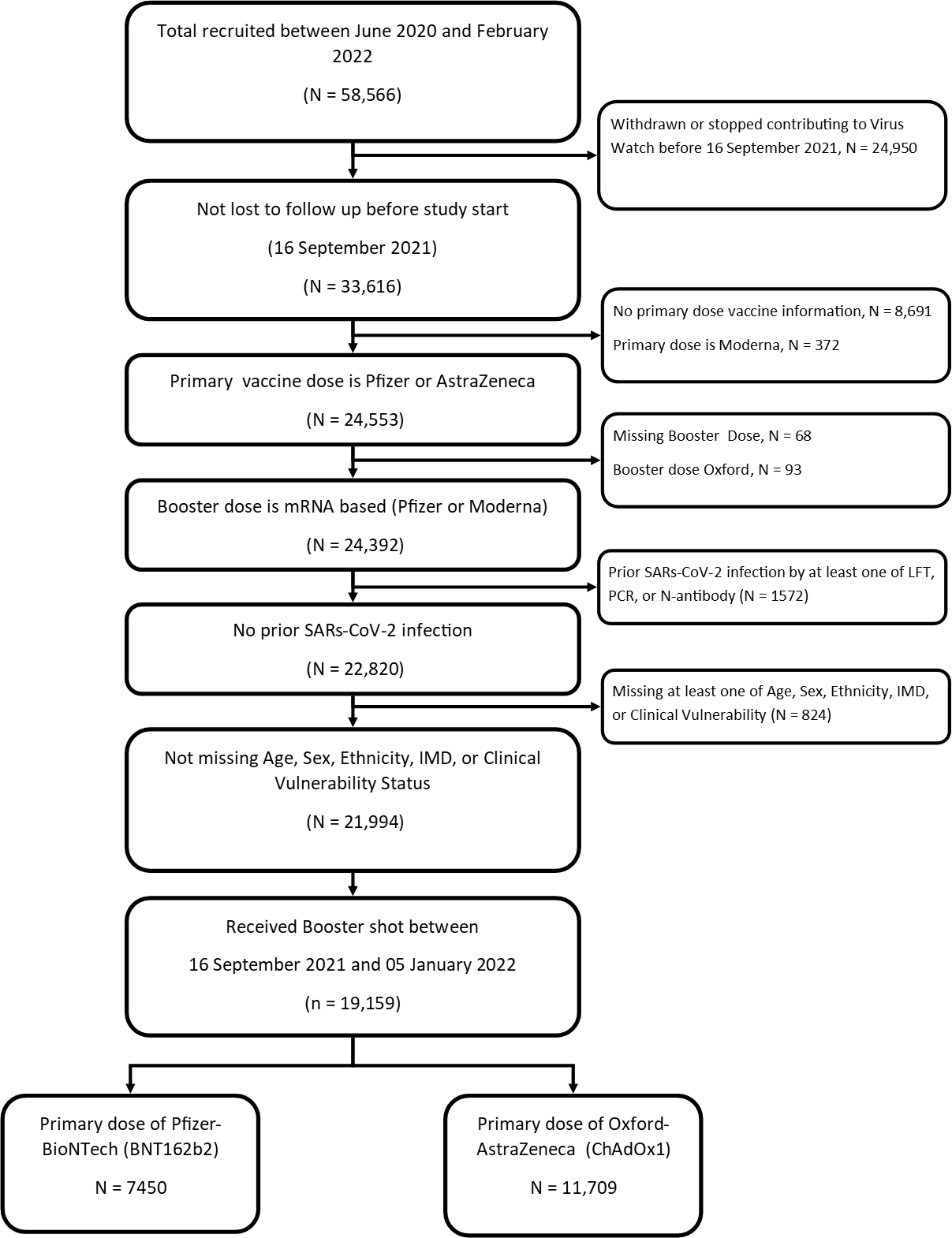


Supplementary Figure S2: Linkage source for the exposure and outcomes for the primary analysis. Percentages are represented as “level” percentages; that is each level sums up to 100%. For Dose 1, 83% of vaccinations were self-reported and 17% were obtained through linkage. For dose 3, 82% of vaccinations were obtained through self-reports, whilst 18% of vaccinations were obtained through linkage. For a positive SARs-CoV-2 infection, 92.05% of infections were obtained through self-reports whilst 7.95% of infections were obtained through linkage.


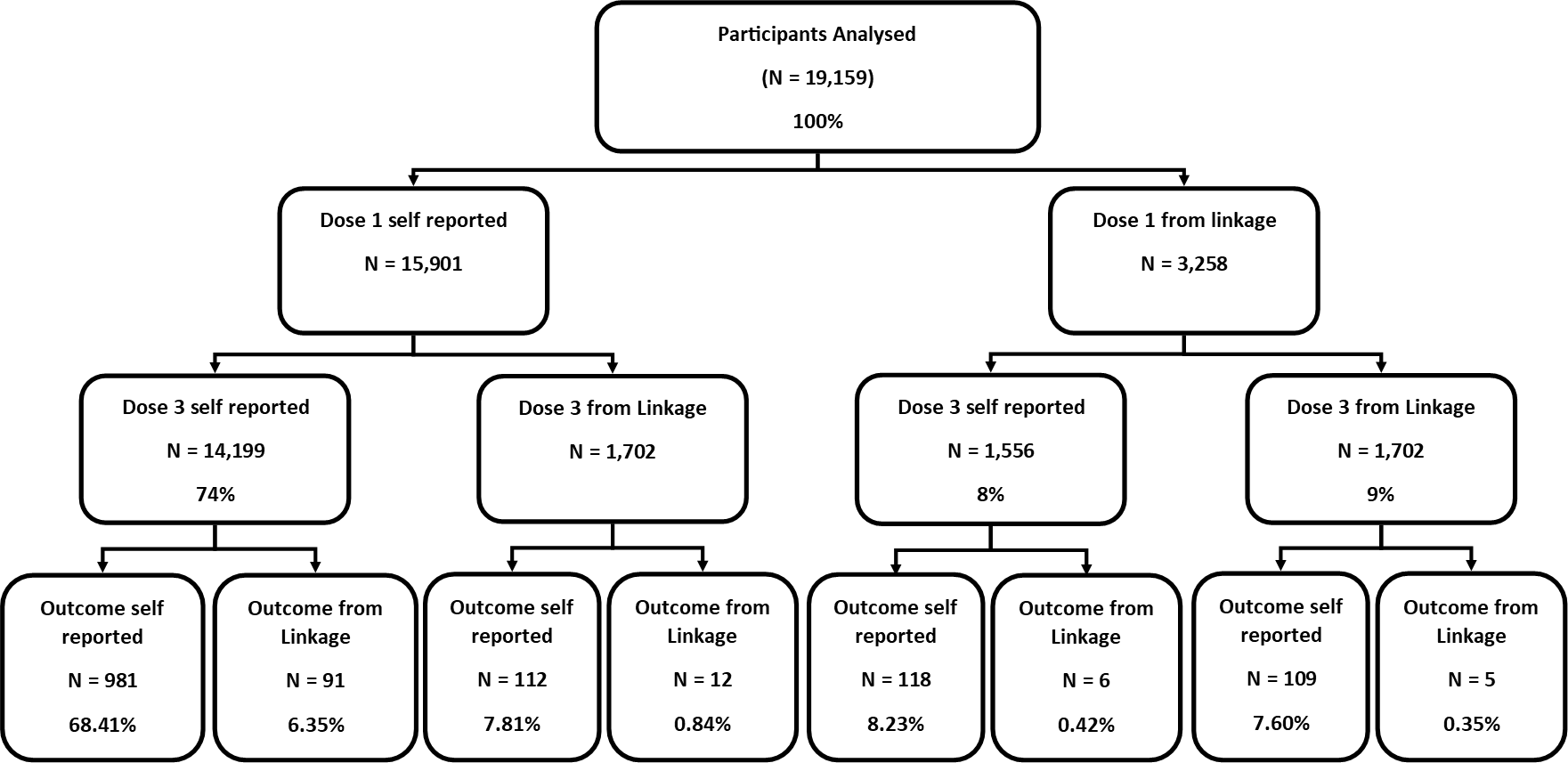


Supplementary Figure S3: Kaplan-Meier curve for all cohorts that compares the incidence of severe acute respiratory syndrome coronavirus 2 (SARS-CoV-2) infections between Oxford-AstraZeneca (ChAdOx1) primary dose individuals against Pfizer- BioNTech(BNT162b2) primary dose individuals

| 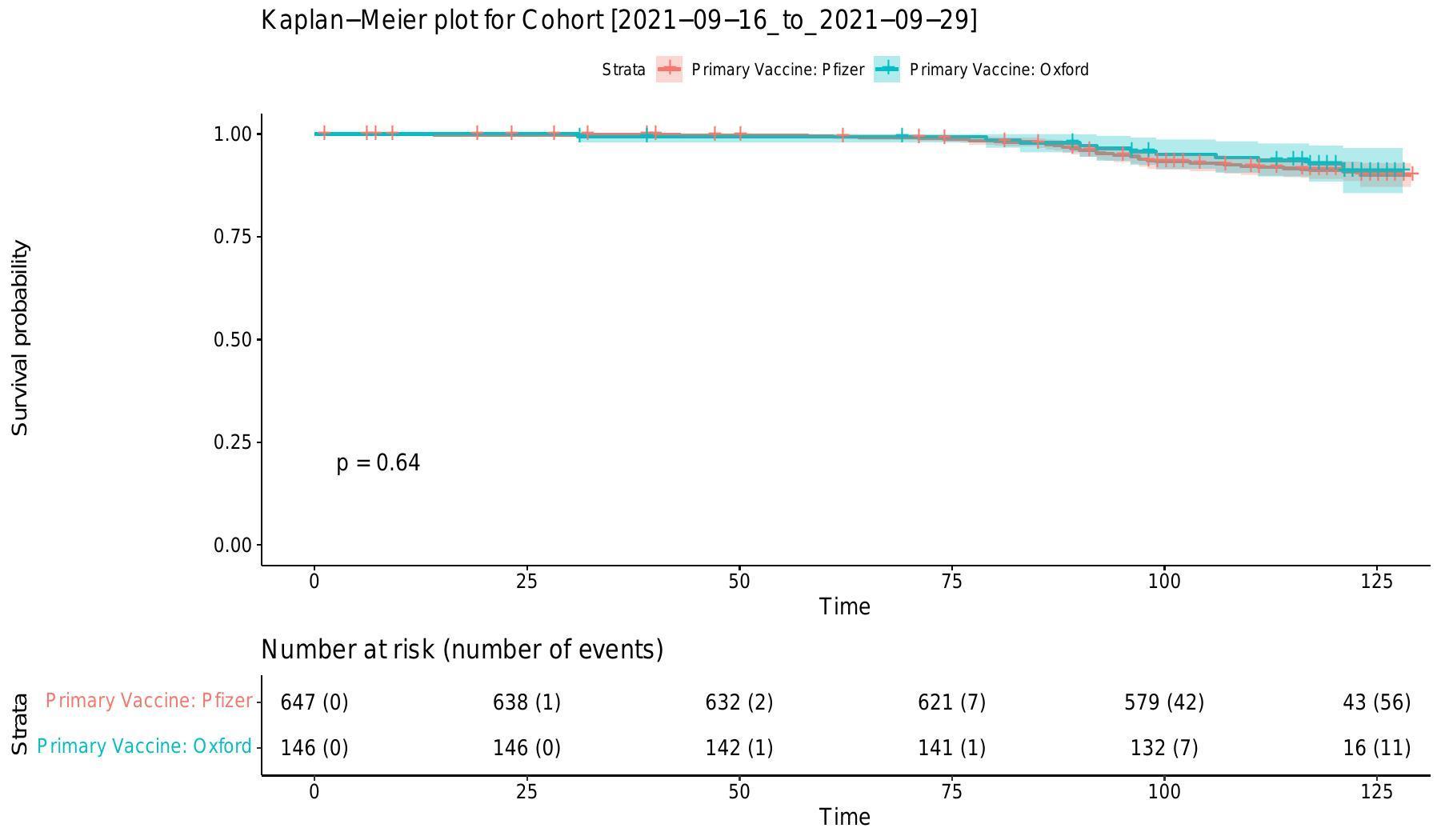 |
| --- |
| 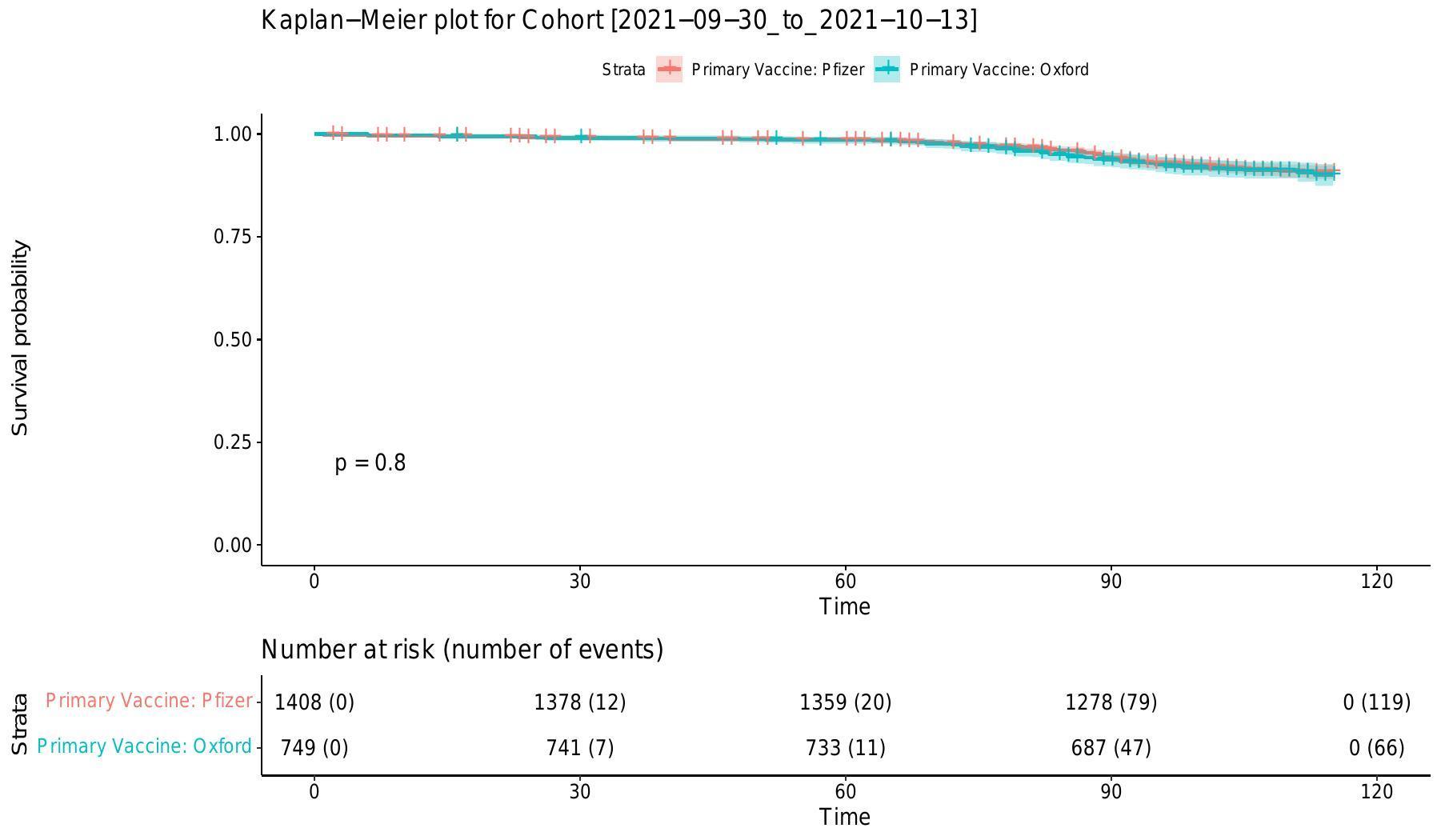 |
| 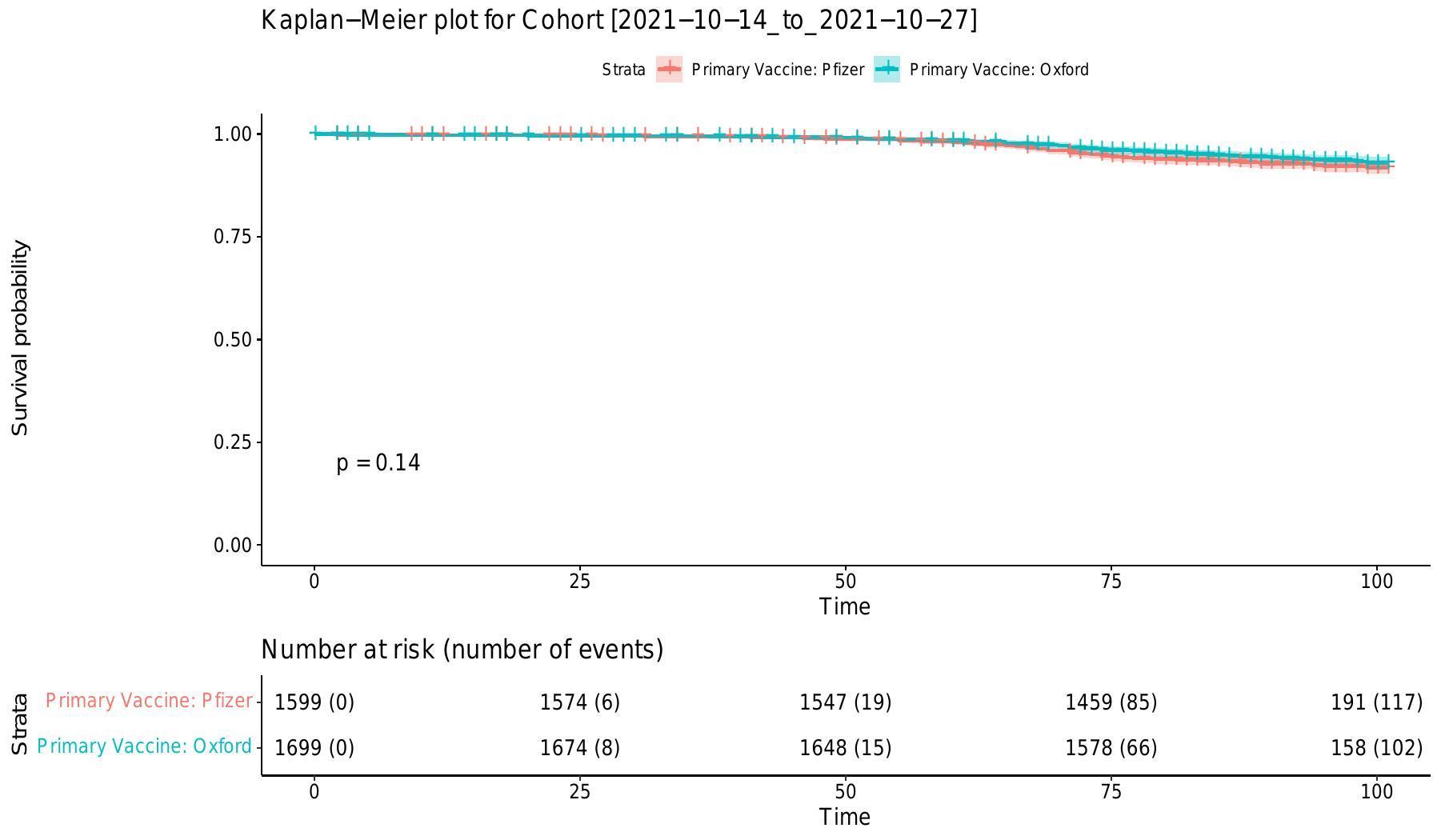 |
| 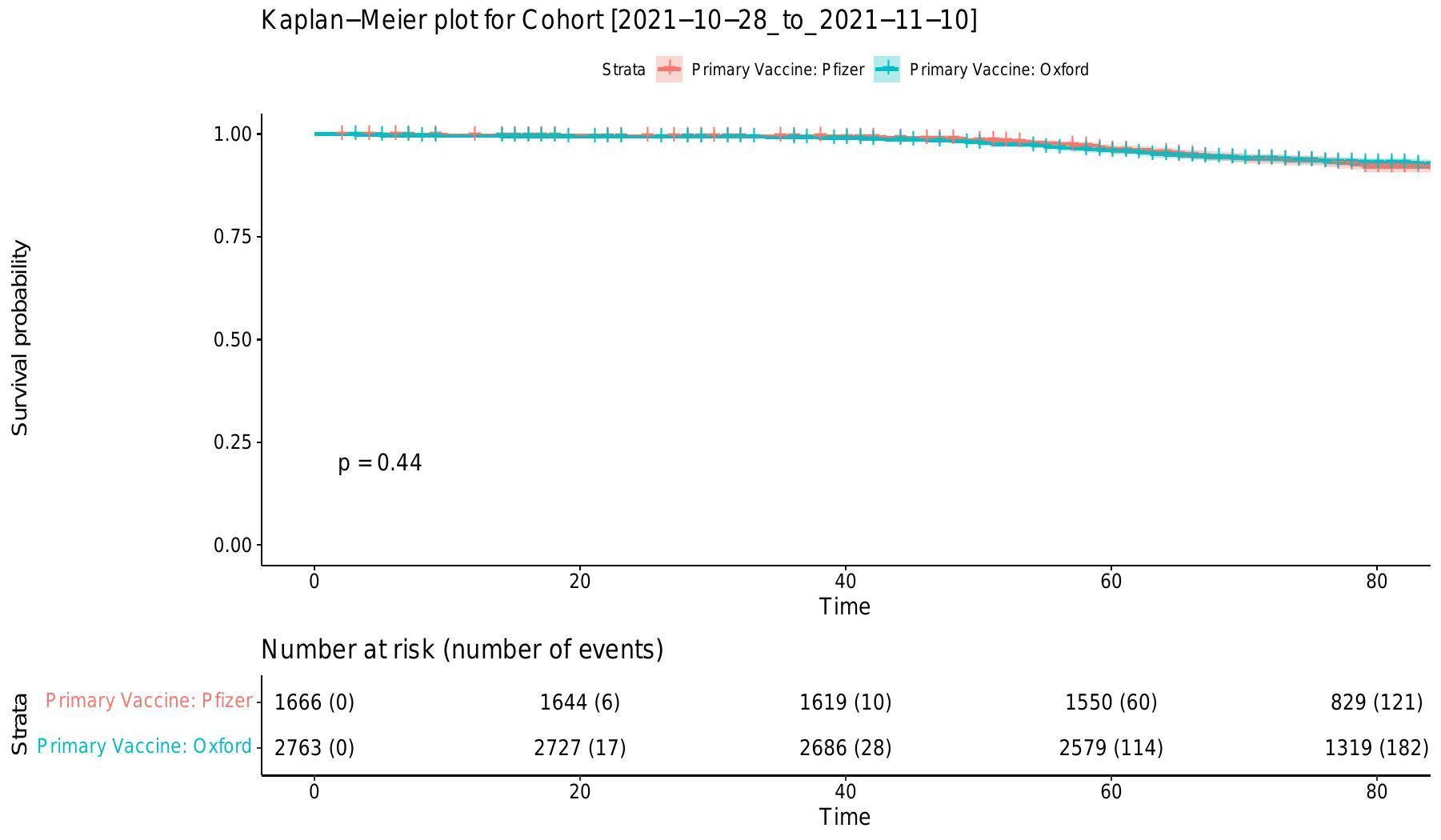 |
| 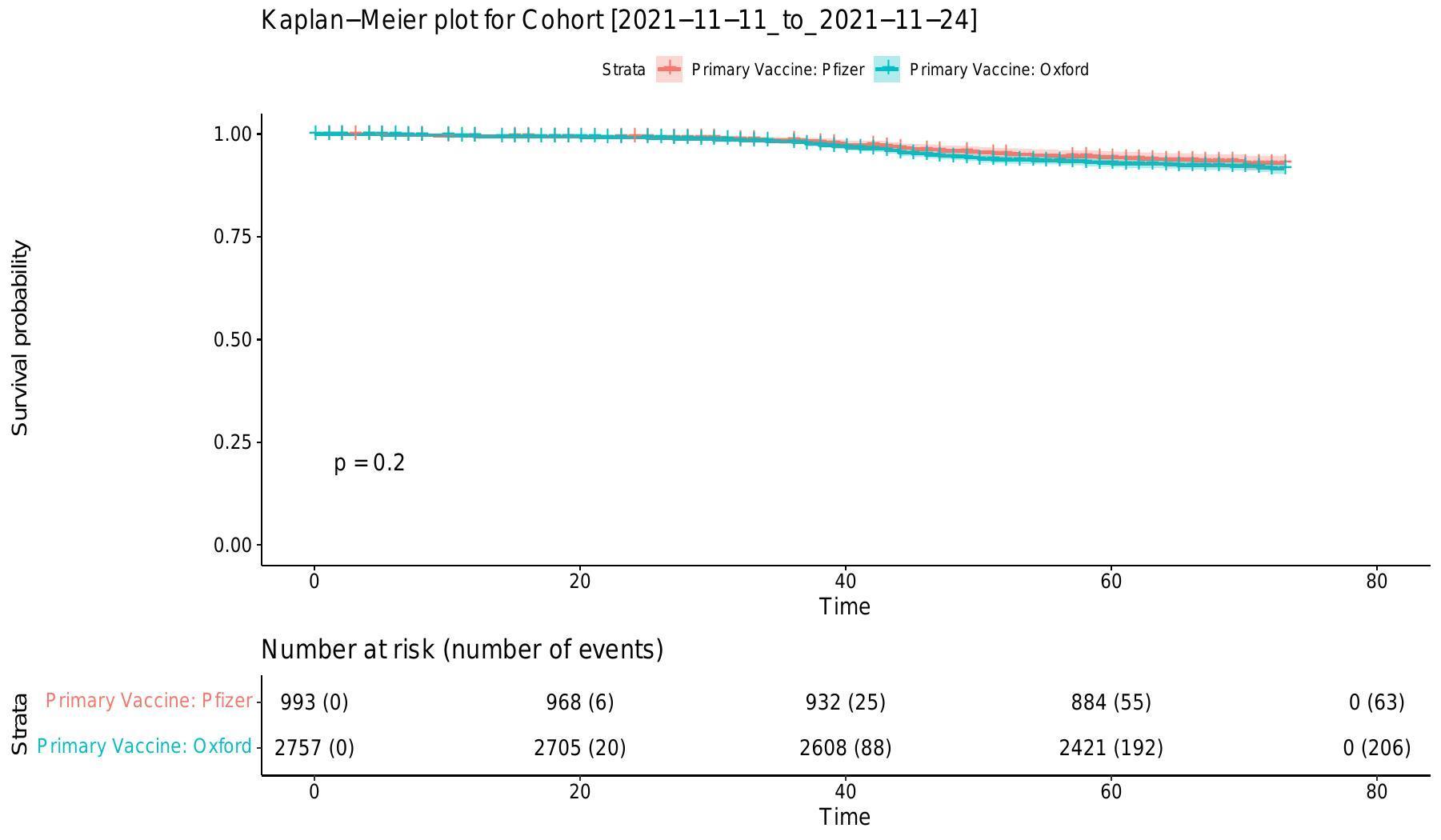 |
| 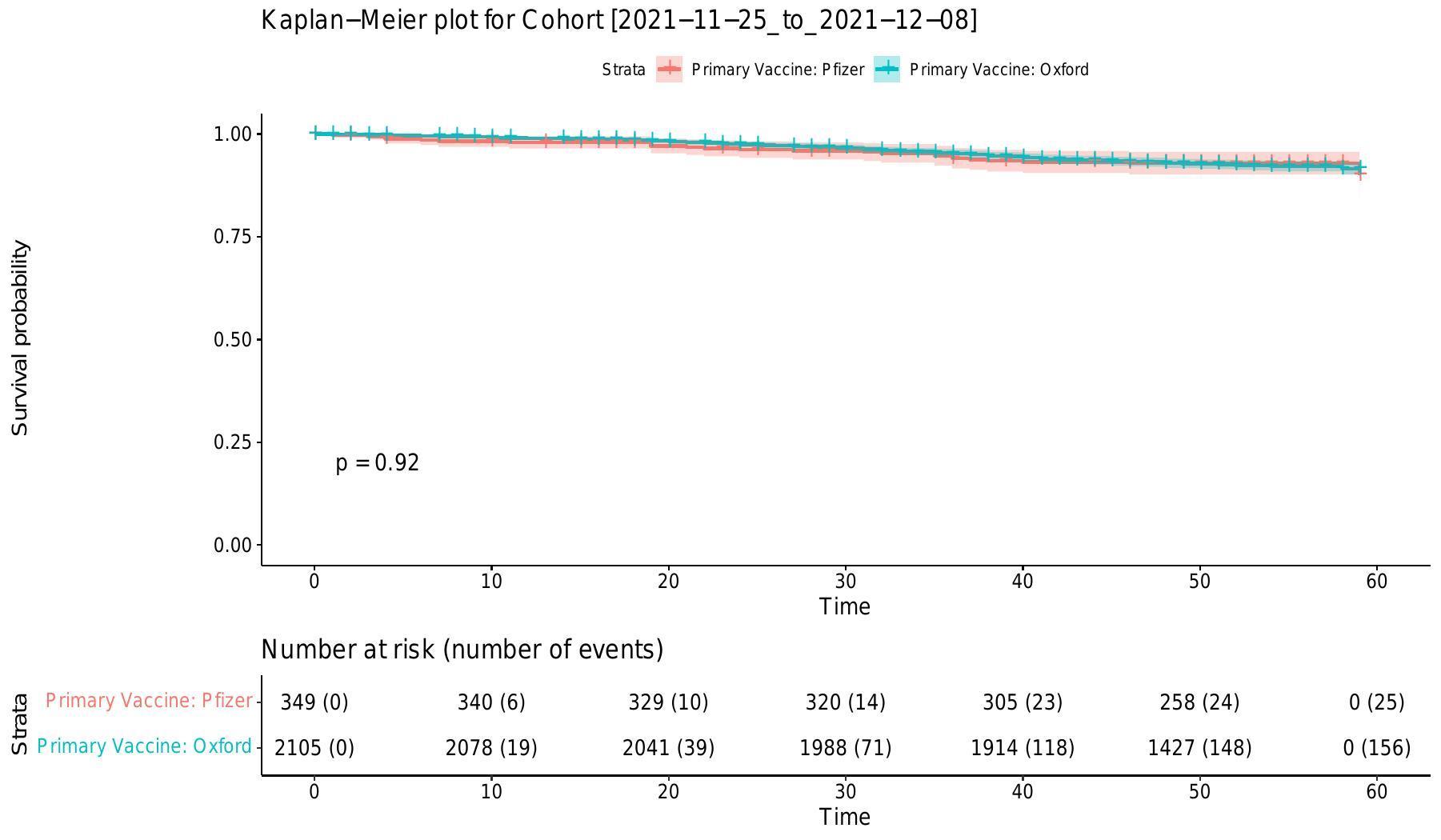 |
| 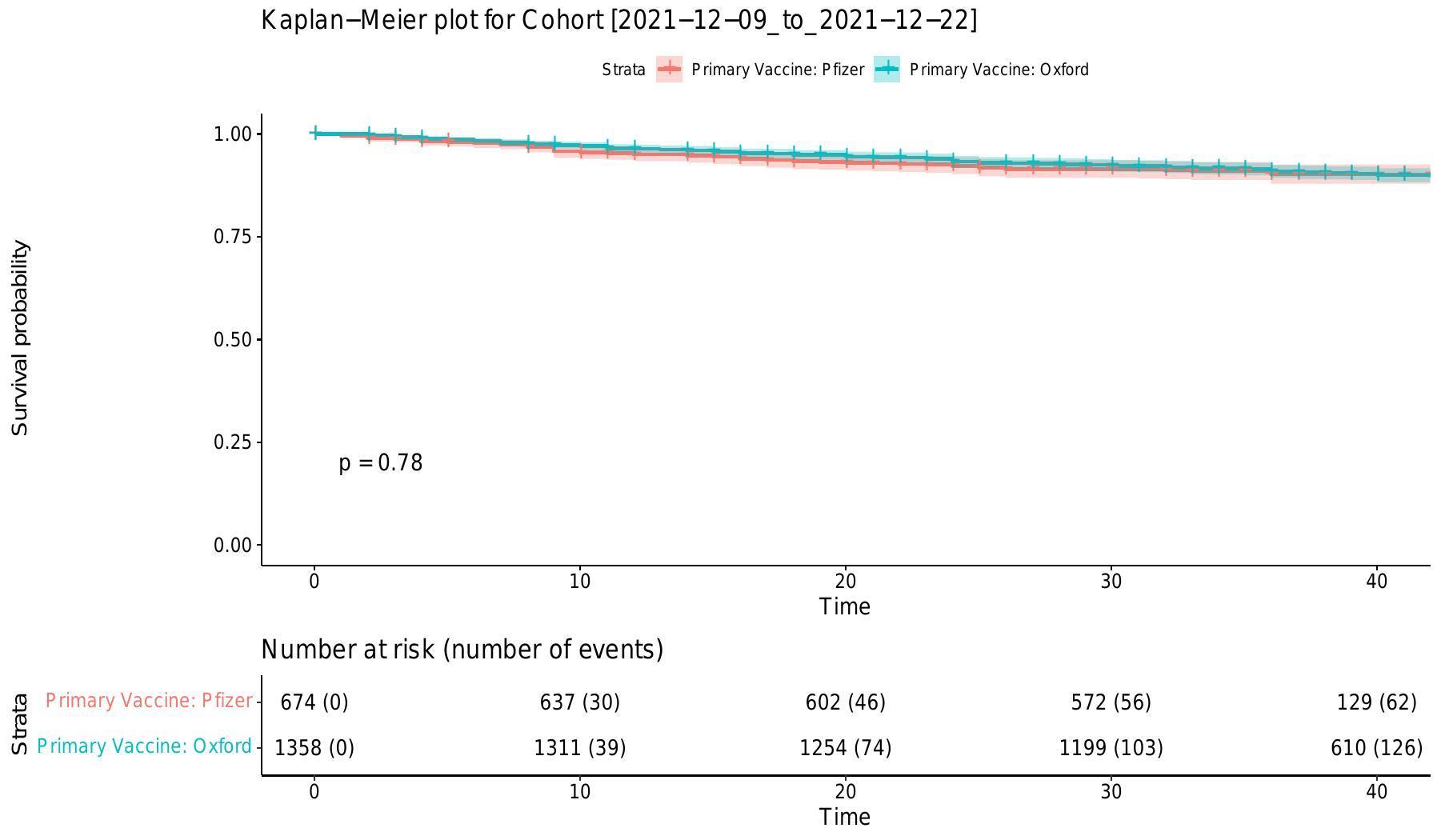 |
| 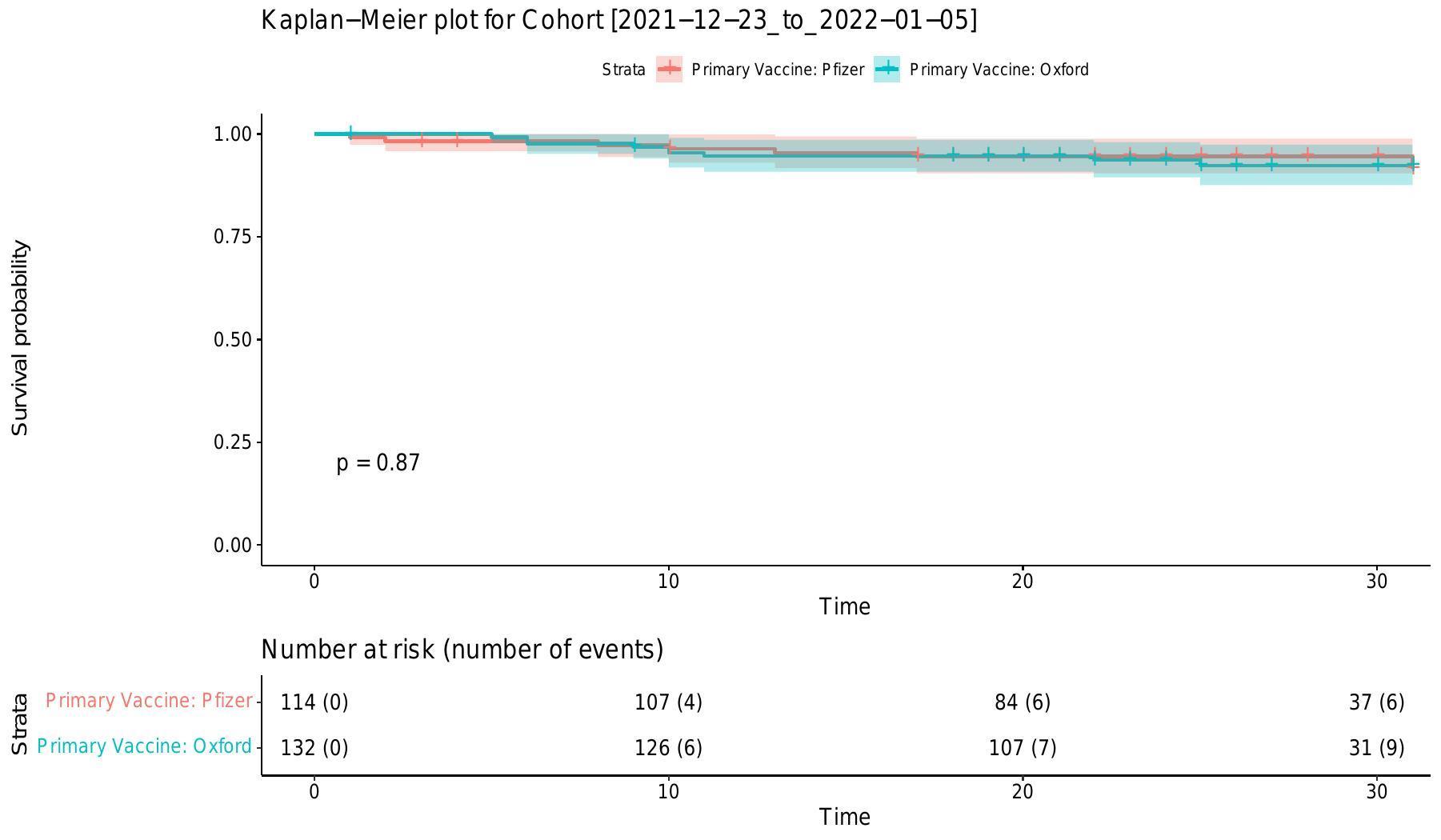 |

Supplementary Figure S4:  Adjusted and unadjusted random-effects meta-analysis for each covariate for the sensitivity analysis where we did not exclude those with a prior severe acute respiratory syndrome coronavirus 2 (SARs-CoV-2) infection. In this figure, logHR represents the natural log of the hazard ratio, SE(logHR) represent the standard error of the hazard ratio, HR represents the hazard ratio, and 95% CI represent the 95% confidence interval.

| **Adjustment** | **Covariate** | **Random Effects Meta-analysis for each covariate** |
| --- | --- | --- |
| Unadjusted | Vaccine type - Oxford AstraZeneca ChAdOx1 in reference to Pfizer BioNTech BNT162b2 | 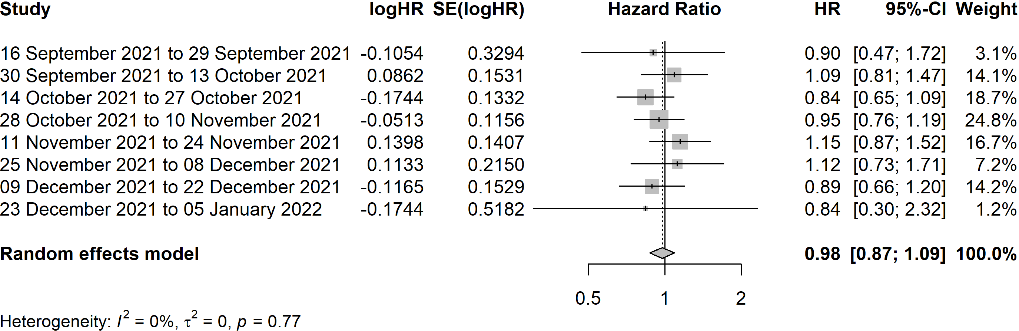 |
| Adjusted | Vaccine type - Oxford AstraZeneca ChAdOx1 in reference to Pfizer BioNTech BNT162b2 | 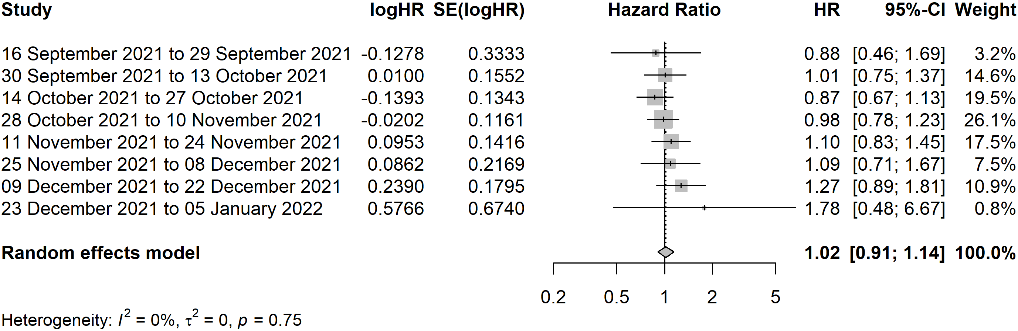 |

Supplementary Figure S5: Adjusted and unadjusted random-effects meta-analysis for vaccination as part of sensitivity analysis two, where we restricted the booster dose to BNT162b2 only. In this figure, logHR represents the natural log of the hazard ratio, SE(logHR) represent the standard error of the hazard ratio, HR represents the hazard ratio, and 95% CI represent the 95% confidence interval.

| **Adjustment** | **Covariate** | **Random Effects Meta-analysis for each covariate** |
| --- | --- | --- |
| Unadjusted | Vaccine type - Oxford AstraZeneca ChAdOx1 in reference to Pfizer BioNTech BNT162b2 | 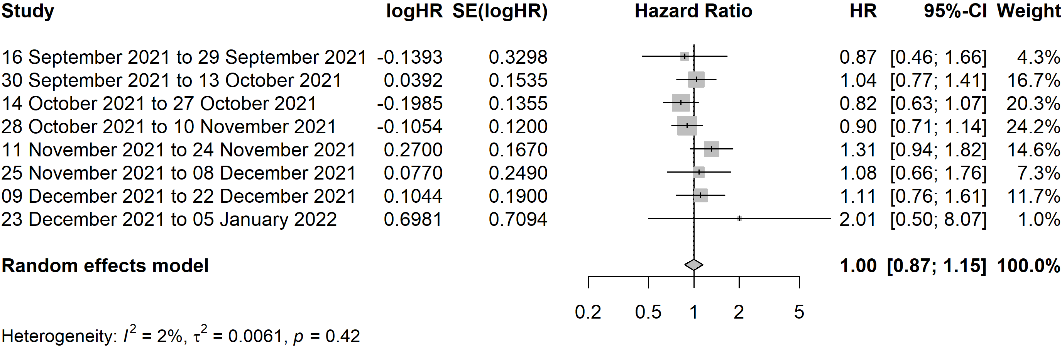 |
| Adjusted | Vaccine type - Oxford AstraZeneca ChAdOx1 in reference to Pfizer BioNTech BNT162b2 | 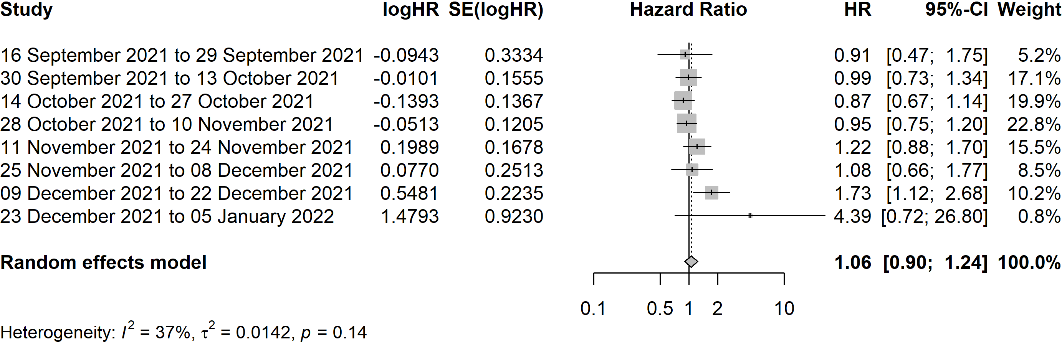 |

Supplementary Figure S6: Adjusted and unadjusted random-effects meta-analysis for vaccination as part of sensitivity analysis three, where we restricted the cohort to those who were successfully linked by NHS digital. In this figure, logHR represents the natural log of the hazard ratio, SE(logHR) represent the standard error of the hazard ratio, HR represents the hazard ratio, and 95% CI represent the 95% confidence interval.

| **Adjustment** | **Covariate** | **Random Effects Meta-analysis for each covariate** |
| --- | --- | --- |
| Unadjusted | Vaccine type - Oxford AstraZeneca ChAdOx1 in reference to Pfizer BioNTech BNT162b2 | 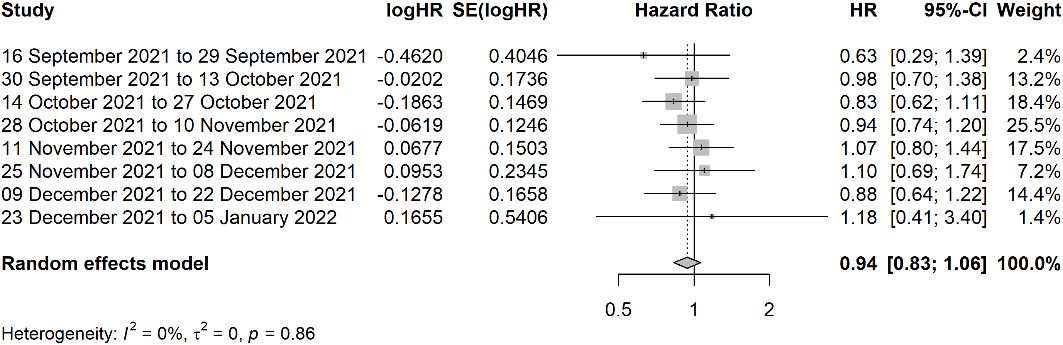 |
| Adjusted | Vaccine type - Oxford AstraZeneca ChAdOx1 in reference to Pfizer BioNTech BNT162b2 | 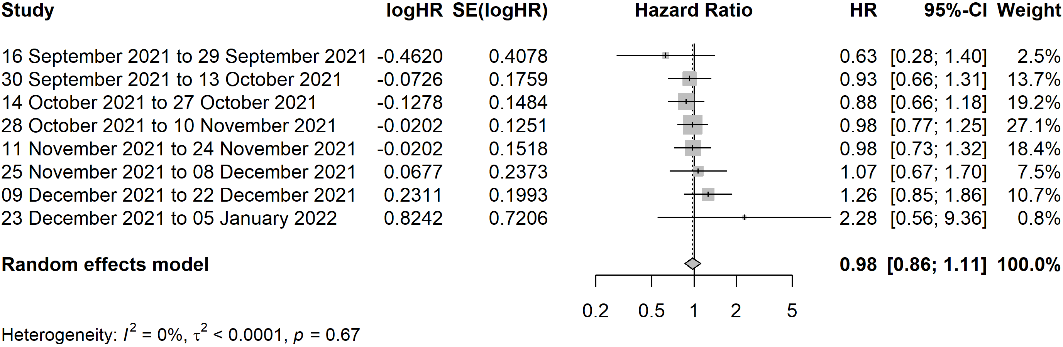 |

Supplementary Figure S7: Adjusted and unadjusted random-effects meta-analysis for vaccination as part of sensitivity analysis four, where the start of follow-up was 8 days after the recorded booster vaccination date to account for the time it takes for antibody development. In this figure, logHR represents the natural log of the hazard ratio, SE(logHR) represent the standard error of the hazard ratio, HR represents the hazard ratio, and 95% CI represent the 95% confidence interval.

| **Adjustment** | **Covariate** | **Random Effects Meta-analysis for each covariate** |
| --- | --- | --- |
| Unadjusted | Vaccine type - Oxford AstraZeneca ChAdOx1 in reference to Pfizer BioNTech BNT162b2 | 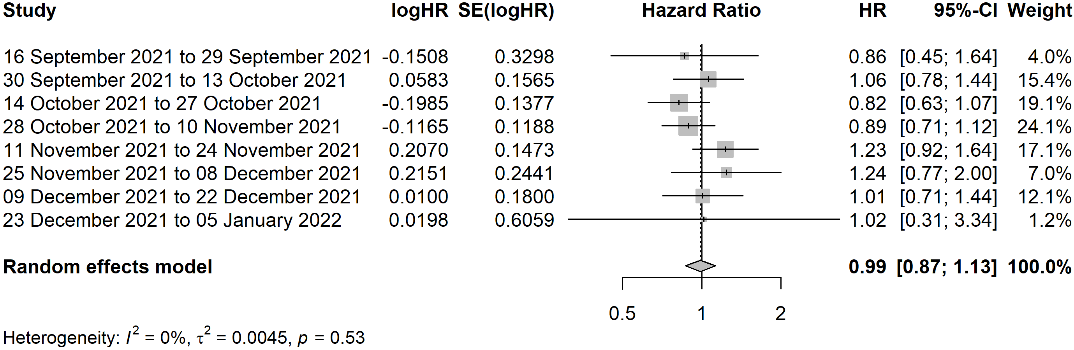 |
| Adjusted | Vaccine type - Oxford AstraZeneca ChAdOx1 in reference to Pfizer BioNTech BNT162b2 | 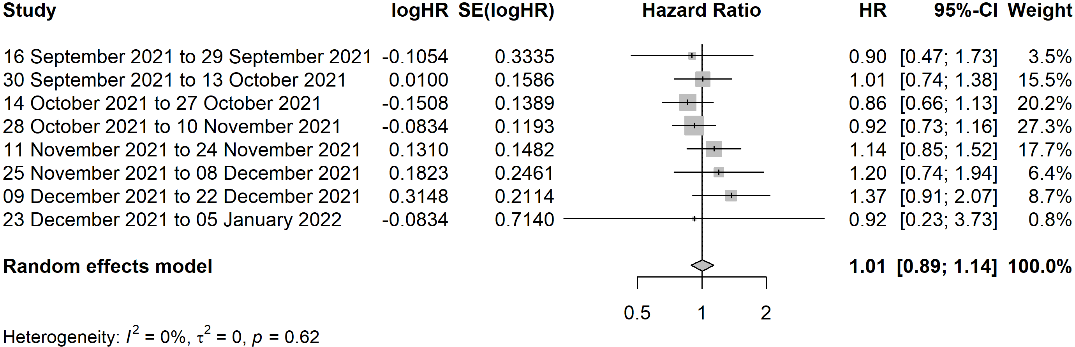 |

Supplementary Figure S8: Vaccination counts by age group at Booster vaccination through time.


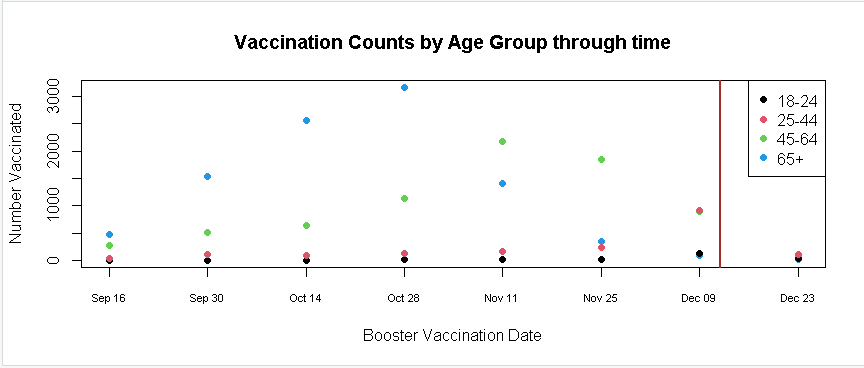

Supplement: dyad002_Supplementary_Data [file dyad002_supplementary_data.docx]
